# Antibacterial Titanium Implants Biofunctionalized by Plasma Electrolytic Oxidation with Silver, Zinc, and Copper: A Systematic Review

**DOI:** 10.3390/ijms22073800

**Published:** 2021-04-06

**Authors:** Ingmar A. J. van Hengel, Melissa W. A. M. Tierolf, Lidy E. Fratila-Apachitei, Iulian Apachitei, Amir A. Zadpoor

**Affiliations:** Department of Biomechanical Engineering, Faculty of Mechanical, Maritime, and Materials Engineering, Delft University of Technology, 2628 CD Delft, The Netherlands; melissa_tierolf@hotmail.com (M.W.A.M.T.); e.l.fratila-apachitei@tudelft.nl (L.E.F.-A.); i.apachitei@tudelft.nl (I.A.); a.a.zadpoor@tudelft.nl (A.A.Z.)

**Keywords:** plasma electrolytic oxidation, additive manufacturing, titanium bone implants, antibacterial biomaterials, surface biofunctionalization, implant-associated infection

## Abstract

Patients receiving orthopedic implants are at risk of implant-associated infections (IAI). A growing number of antibiotic-resistant bacteria threaten to hamper the treatment of IAI. The focus has, therefore, shifted towards the development of implants with intrinsic antibacterial activity to prevent the occurrence of infection. The use of Ag, Cu, and Zn has gained momentum as these elements display strong antibacterial behavior and target a wide spectrum of bacteria. In order to incorporate these elements into the surface of titanium-based bone implants, plasma electrolytic oxidation (PEO) has been widely investigated as a single-step process that can biofunctionalize these (highly porous) implant surfaces. Here, we present a systematic review of the studies published between 2009 until 2020 on the biomaterial properties, antibacterial behavior, and biocompatibility of titanium implants biofunctionalized by PEO using Ag, Cu, and Zn. We observed that 100% of surfaces bearing Ag (Ag-surfaces), 93% of surfaces bearing Cu (Cu-surfaces), 73% of surfaces bearing Zn (Zn-surfaces), and 100% of surfaces combining Ag, Cu, and Zn resulted in a significant (i.e., >50%) reduction of bacterial load, while 13% of Ag-surfaces, 10% of Cu-surfaces, and none of Zn or combined Ag, Cu, and Zn surfaces reported cytotoxicity against osteoblasts, stem cells, and immune cells. A majority of the studies investigated the antibacterial activity against *S. aureus*. Important areas for future research include the biofunctionalization of additively manufactured porous implants and surfaces combining Ag, Cu, and Zn. Furthermore, the antibacterial activity of such implants should be determined in assays focused on prevention, rather than the treatment of IAIs. These implants should be tested using appropriate in vivo bone infection models capable of assessing whether titanium implants biofunctionalized by PEO with Ag, Cu, and Zn can contribute to protect patients against IAI.

## 1. Introduction

Implant-associated infections (IAI) are a devastating complication for patients receiving bone implants in total joint arthroplasty, trauma surgeries, and malignant bone tumor resections [1,2,3]. These infections form a tremendous burden for both patients and society. As the number of implantations continues to grow annually [4,5,6], the need for a cure increases. Given that the treatment of such infections is highly costly from both financial and societal points of view, the focus has shifted towards the prevention of IAI through the development of implants with intrinsic antibacterial activity.

Antibiotics form the primary source of antibacterial agents used to treat bacterial infections. However, a vast number of IAI is caused by *Staphylococci* and multiple strains have developed high levels of antibiotic resistance [7,8], raising concerns for the future treatments of IAI. Infection by methicillin-resistant *Staphylococcus aureus* (MRSA) highly complicates the treatment of IAI and adversely affects the treatment outcomes [9,10]. Other antibacterial agents are, therefore, being investigated. Metallic elements, such as Ag, Cu, and Zn have shown strong antibacterial behavior against a wide microbial spectrum, including resistant bacterial strains [11,12,13,14].

Ag has excellent antibacterial properties, but may also induce cytotoxicity [15,16]. Cu and Zn, on the other hand, exhibit lower levels of antibacterial behavior, but are essential trace elements. Furthermore, they have been found to enhance the cytocompatibility of implant surfaces [17,18]. Therefore, combining these elements may result in the right balance between antibacterial behavior, chemical biocompatibility, and osteogenic response [19,20]. 

The local administration of antibacterial agents at the implant site was shown to greatly complement the systemic administration of antibiotics [21,22]. The side effects of such agents can also be prevented as the required antibacterial dose is generally lower [23]. To deliver antibacterial agents locally, the surface of the implants can be biofunctionalized through surface treatment techniques. Antibacterial agents can be attached to implants either as a coating layer, embedded directly onto the implant surface, or incorporated as part of a converted surface layer [24]. 

Antibacterial agents can be deposited onto the implant surface by means of polymeric, ceramic or metallic coatings. To produce these coatings, usually low temperatures are used and therefore little interaction occurs with the implant substrate. Coatings have a tendency to be thin and fragile, thereby limiting the availability of the antibacterial agent and hampering their use during surgical implantation. To enhance the diffusion, the antibacterial agent can be incorporated in a biodegradable polymer coating. In this way implants were manufactured that contain Ag [25,26], Cu [27], and Zn [28]. Polymeric coatings can be attached onto an implant by dipping and drying, sol-gel technology, spray drying, layer-by-layer manufacturing, and self-assembly monolayers. Downsides are the limited mechanical and chemical stability, local inflammatory response due to degradation products, and uncontrolled release kinetics.

Another strategy is direct embedding of the antibacterial agent into the implant surface. In this way, no new material is added on top of the substrate, but the composition of the outermost layer of the implant substrate is altered. Examples of such methods are ion implantation, plasma immersion ion implantation [29], and in situ reduction [30]. Advantages are that the implant surface morphology remains intact, and the corrosive and biocompatible properties of the substrate material retained. However, this strategy is difficult to perform on complex geometries and does not allow for optimization of the surface morphology.

A third approach to incorporate Ag, Cu, and Zn in the implant surface is through generation of a converted surface layer. One such technique is plasma electrolytic oxidation (PEO), which was investigated to biofunctionalize the surface of highly porous implants made of specific metallic biomaterials [31]. During PEO, the native titanium oxide layer is transformed into a crystalline and microporous surface in a swift and single-step process. 

Through the addition of antibacterial elements into the PEO electrolyte, these elements become part of the converted surface layer and result in a surface exhibiting antibacterial behavior [32,33]. Due to the tight embedding of the antibacterial agents into the surface, the release of these ions can be controlled and the undesired circulation of agents can be prevented, thereby avoiding nanotoxic effects [34]. PEO was applied to generate titanium implants with antibacterial properties using Ag, Cu, and Zn [35,36,37]. In addition to the antibacterial behavior, PEO biofunctionalized surfaces were shown to enhance osseointegration and stimulate bony ingrowth in vivo [38,39].

Bone implants are increasingly produced through additive manufacturing (AM), as this allows free-form fabrication and customized treatment for patients. AM allows for the fabrication of highly porous implants with vast internal surface areas, which may make the implants more prone to infection, while at the same time providing a challenging surface to modify through surface biofunctionalization techniques. PEO is capable of biofunctionalizing the surface of complex geometries. In addition, the parameters of the PEO process can be controlled, which allows to tailor the chemistry of the surface layer [40,41]. Furthermore, the synthesized surface layer adheres strongly to the implant substrate. Moreover, the method is easily scalable towards clinically sized implants. Limitations of PEO are that the surface morphology and chemistry of the surface are modified simultaneously and this makes the individual tuning of these properties difficult. Furthermore, the exact mechanism of plasma discharging is still unknown, and thereby the fine-tuning of the PEO processing parameters difficult to predict [42]. 

In order to develop clinically relevant antibacterial implants, it is important to assess the progress made in this area and compare the outcomes of different studies. As most implants available for current clinical use are made of titanium, we performed a systematic review on titanium implants biofunctionalized by PEO using Ag, Cu, and Zn. In order to illustrate the progress made in this area, we screened the studies published between 2009 and December 2020. This area of research involved several scientific disciplines, including engineering, material sciences, microbiology, and orthopedics. We, therefore, analyzed a broad spectrum of aspects including the implant substrate, PEO parameters, surface characteristics, antibacterial assays, and cytocompatibility testing. 

## 2. Methods

### 2.1. Literature Search

A comprehensive electronic search was performed using Scopus and Google Scholar search engines up until December 2020. In addition, a global screening was performed using PubMed. The article search was conducted using different combinations of the following keywords: plasma electrolytic oxidation, micro-arc oxidation, antibacterial activity, Ag, Cu, and Zn. To ensure that relevant publications were not excluded, combinations of subject headings, text-word terms, and the Boolean operators AND and OR were used. The searches were limited to those studies published in English between 2009 and 2020. The reference lists of the included eligible studies were scanned to ensure no eligible studies were omitted. The last search date was 24 December, 2020. This systematic review was written according to the PRISMA (Preferred Reporting Items for Systematic Review and Meta-Analyses) statement [43].

### 2.2. Inclusion and Exclusion Criteria

The inclusion criteria were—(1) the surface modification technique: plasma electrolytic oxidation (PEO), micro-arc oxidation (MAO), or anodic spark deposition (ASD); (2) implant substrate: titanium and its alloys; (3) antibacterial agents: Ag, Cu and Zn; (4) metallic-based antibacterial agents should have been incorporated in PEO-modified Ti-based surfaces; and (5) assessment of the antibacterial behavior should have been performed. A study was excluded if it did not report any outcome variable. Furthermore, studies were not eligible for inclusion when—(1) articles were not published in English; (2) no surface modification technique was utilized; (3) PEO was performed in combination with other surface modification techniques or treatments; (4) no antibacterial testing was performed; and (5) study was of one of the following document types: reviews, patents, conference abstracts/papers, and case reports.

### 2.3. Study Selection

The titles and abstracts were screened to assess the suitability of the search results. Subsequently, the full-text of the studies selected in the first stage of screening were analyzed to assess whether or not they satisfied the inclusion criteria. 

### 2.4. Risk of Bias

The methodological details of the included studies were analyzed to minimize the risks of biases in the individual studies. Furthermore, excluding grey literature in Google Scholar decreased the risk of biases in the evaluation.

### 2.5. Data Extraction

Extracted information included the type of the titanium substrate, electrolyte composition, PEO processing parameters, surface topography, XRD phase composition, surface content of the incorporated elements, the release profile of the metallic (i.e., Ag, Cu, and Zn) ions, antibacterial assays, tested pathogens, eukaryotic cell types, and the outcomes (i.e., antibacterial behavior and cytocompatibility). The results were considered significant when *p* < 0.05.

### 2.6. Search Results

**Figure 1:**
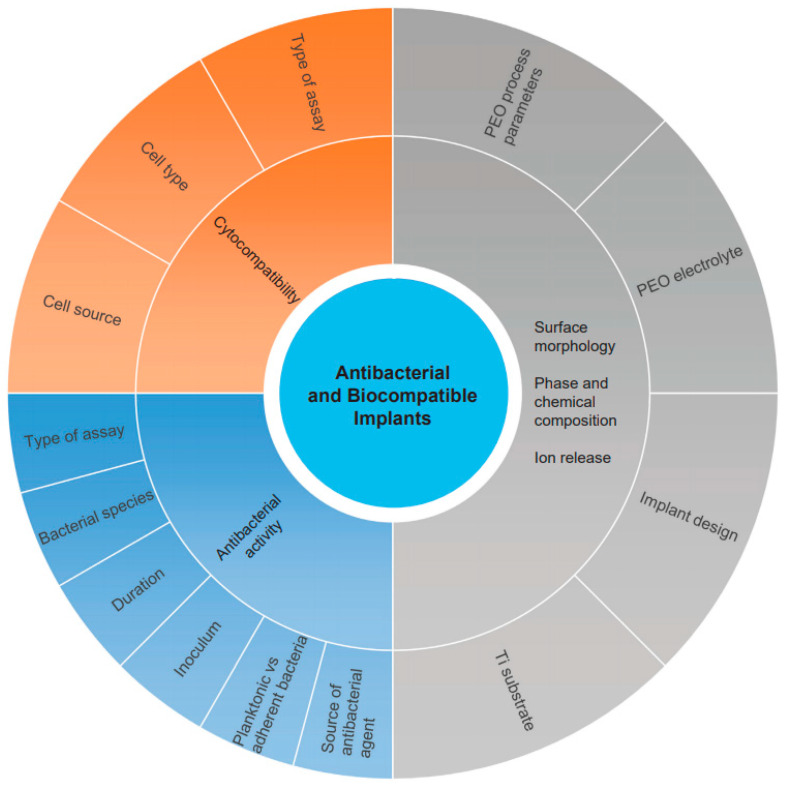
A graphical presentation of the outline of this systematic review.

A total of 1261 studies were identified in the two search engines: 1190 from Google Scholar and 71 from Scopus. After screening the titles and abstracts, 1158 studies were excluded. The primary reasons for exclusion were no antibacterial or biocompatibility tests, PEO performed in combination with other surface modification techniques, and document types: reviews, patents, conference abstracts/papers, citations and case reports. As a result, 103 studies were selected for full-text analysis. The analysis led to the exclusion of 59 studies, as they failed to meet the inclusion criteria. Finally, 49 studies were included in this systematic review and were used for a qualitative analysis of their data and for comparison with each other. A flow diagram was created to represent the entire systematic search of the relevant studies (Supplementary Figure S1). The outline of the review is presented in Figure 1.

## 3. Summary of Study Characteristics

**Figure 2:**
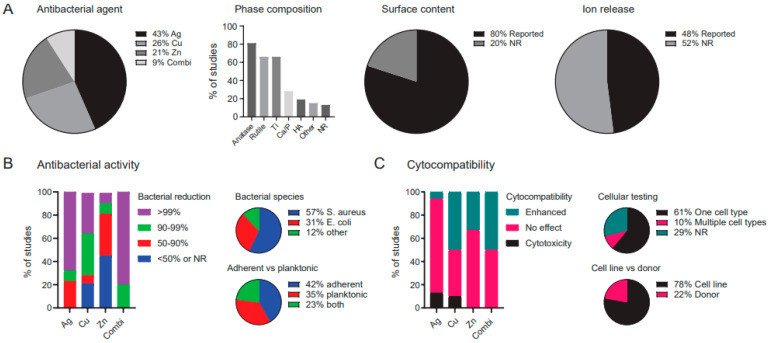
An overview of the (**A**) biomaterial, (**B**) antibacterial, and (**C**) cytocompatibility specifications of the studies included in this systematic review of the literature. Combi: combination of Ag, Cu, and/or Zn, HA: hydroxyapatite, NR: not reported.

A summary of the study characteristics is presented in Figure 2. Of the analyzed studies, 43% used Ag, 26% used Cu, and 21% worked with Zn, while 9% investigated a combination of Ag, Cu, and Zn (i.e., using two or more metallic agents. Various types of parameters were reported in the studies (Figure 2A), including the PEO processing parameters (98%), phase composition (87%), surface content of the incorporated elements (80%), and ion release kinetics (48%). Furthermore, 92% of the studies quantified the antibacterial activity, which was reported to be >50% for 100% of the studies using Ag, 93% of the studies using Cu, and 73% of those employing Zn, as well as 100% of the studies combining multiple metallic agents (Figure 2B). Of those studies, 57% tested the efficacy of the surfaces against *S. aureus*, 31% of the studies tested their specimens against *E. coli,* while 12% of the studies chose other bacterial species. Furthermore, the antibacterial activity was determined against adherent bacteria in 42% of the studies, while 35% of the studies assessed the antibacterial activity of their specimens against planktonic bacteria, and 23% assessed both.

Cytocompatibility was tested in 71% of all studies, of which 10% tested against multiple cell types (Figure 2C). Of the studies assessing the cytocompatibility of their specimens, 78% used a cell line while 22% used cells obtained from a donor. The addition of the metallic antibacterial agent resulted in cytotoxicity for 13% of the Ag studies, 10% of the Cu studies, 0% of the Zn studies, and 0% of the studies combining two or more metals. Meanwhile, improved cell response (i.e., enhanced cell viability and/or osteogenic differentiation) was observed for 7% of the Ag surfaces, 50% of the Cu surfaces, and 33% of the Zn surfaces, as well as for 50% of the surfaces combining Ag, Cu, and Zn.

## 4. Synthesis and Characterization of PEO Biofunctionalized Surfaces

**Figure 3:**
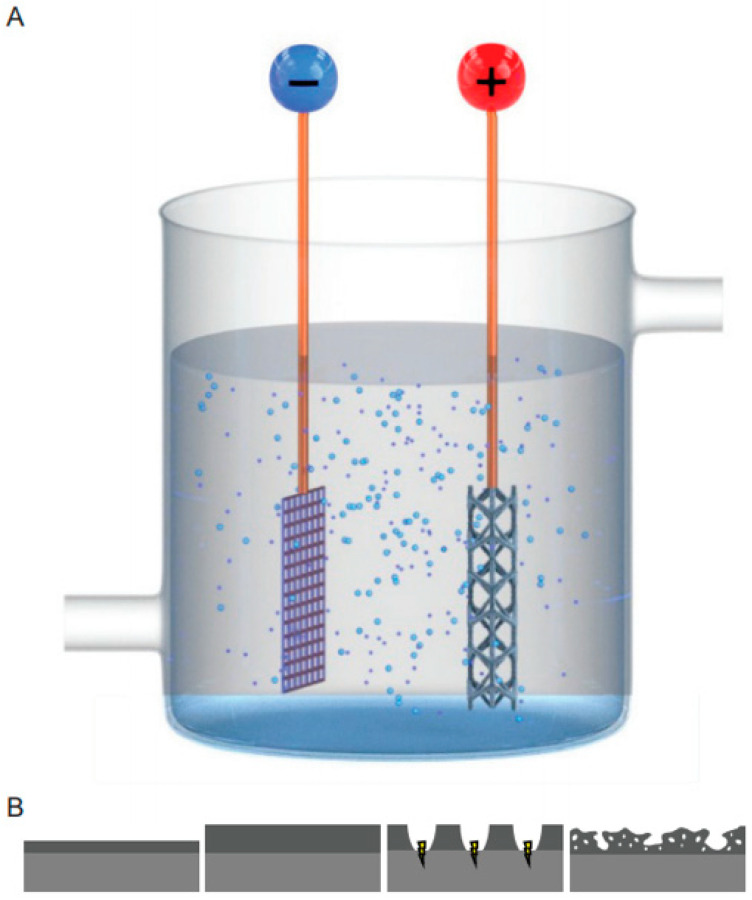
(**A**) A schematic drawing of the plasma electrolytic oxidation (PEO) setup with a cathode and an anode (implant). (**B**) During PEO processing, initially the titanium oxide layer grew outwards. After dielectric breakdown, plasma discharge occurred at the surface, resulting in a highly porous structure.

PEO is an electrochemical process that converts the outer oxide layer of valve metals into a ceramic surface layer and is applied to enhance corrosion resistance [44], dielectric properties [45], and biocompatibility [46] of the substrates. A PEO setup has two electrodes: the cathode and anode (Figure 3A). Usually, either a constant current or voltage is applied, leading to the formation of an oxide layer on the anode (i.e., the specimen to be treated). After dielectric breakdown, the oxide layer is thickened by spark discharges that lead to pore formation [47] (Figure 3B). As the process continues, the sparks become more intense, resulting in the formation of larger pores.

PEO biofunctionalization results in an altered surface morphology and chemical composition. In order to relate the antibacterial activity to certain surface characteristics, the surface of the biofunctionalized specimens is usually characterized (Table 1, Table 2, Table 3 and Table 4). The important surface parameters in this regard are the surface topography, chemical composition, phase composition, and ion release profile. In the following sections, we will discuss the results regarding each of these parameters in more detail.

### 4.1. Titanium Substrate

Of the reviewed studies, most used commercially pure (CP) titanium (62%), followed by Ti6Al4V (23%), Ti6Al7Nb (4%) [15,32], Ti40Nb [52], Ti29Nb13Ta4.6Zr [59], and Ti15Mo [86]. Titanium is used for bone implants because of its mechanical properties, corrosion resistance and chemical biocompatibility [46,89]. Ti6Al4V has a higher strength to weight ratio than CP titanium and is, therefore, the natural choice for load-bearing applications, such as joint replacing implants, while CP titanium is more frequently applied for non-load bearing applications, such as maxillofacial implants [90]. Clinical studies comparing the long-term outcomes of patients treated with either CP-Ti or Ti-alloys are lacking [91,92]. 

Ti6Al4V implants may release vanadium and aluminum ions that can induce cytotoxicity [93]. Other alloys employing niobium have, therefore, been developed, including Ti6Al7Nb and Ti40Nb, which have similar mechanical properties, but do not induce cytotoxicity [94]. In addition, the cytotoxic effects of Al and/or V can be mitigated by PEO, since it reduces the ion release of those species [89]. PEO is easily scalable and can be applied to human-sized implants [95]. In order to translate the results from in vitro studies, it is, therefore, interesting to investigate the antibacterial behavior of substrates that are designed and produced like an implant, for instance, through additive manufacturing. This also highlights one of the advantages of PEO, namely that it can be applied on highly porous surfaces [31].

### 4.2. PEO Electrolyte

The bioactivity of PEO-biofunctionalized implant surfaces is determined for a large part by the composition of the PEO electrolyte, as the elements in the electrolyte eventually make up the chemical composition of the implant surface. More than 50% of the studies included in this systematic review used electrolytes with Ca and P elements. The presence of Ca and P in the electrolyte can result in the formation of hydroxyapatite, which forms more than 60% of bone tissue and is associated with a Ca/P ratio of 1.67 [96,97]. Calcium acetate and calcium glycerophosphate were the primary source of Ca, while CaCO_3_ [52] and C_12_H_22_CaO_14_ [67] were also used in some studies. P is usually added in the form of calcium glycerophosphate, β-glycerophosphate, H_3_PO_4_ [52,69], K_4_P_2_O_7_ [60], NaH_2_PO_4_ [48,49,65,71,74,75], NaPO_3_ [67], or Na_5_P_3_O_10_ [85]. Another element used in about 30% of the included studies is Na in the form of NaOH, NaH_2_PO_4_ [48,49,65,71,74,75], NaPO_3_ [67], Na_5_P_3_O_10_ [85], or Na_2_SiO_3_ [73,76,78,98]. The addition of Na roughens the surface and enhances the Ca/P ratio [99], which has been shown to enhance the osteogenic cell response [100,101]. In addition, the implantation of Na through plasma immersion has been found to stimulate the osteogenic differentiation of cells [102]. Moreover, KOH [60,70,88] is used as an alternative base for NaOH given its similar effects on osteogenic differentiation [103].

### 4.3. PEO Processing Parameters

The electrical parameters of the PEO process affect the surface morphology [42], including the porosity [104], pore size [105], pore shape [106], and pore density [107], as well as the surface chemistry [83,84]. Of the included studies, 54% controlled the voltage, 31% controlled the current density, and 13% controlled both, while 1 study did not report the PEO processing parameters. The oxidation times ranged between 0 and 180 min, with 21% between 0–4 min, 50% between 5–9 min, 19% between 10–14 min, 6% between 15–19 min, and 4% ≥ 20 min. As the current density, voltage, or oxidation time increases, the spark discharge energy amplifies, affecting the mass of the oxide layer formed by a single pulse and resulting in enhanced growth of the oxide layer [40,108]. Furthermore, as temperature of the local discharge area increases, the plasma effect is enhanced, resulting in larger pore sizes and the transformation of amorphous TiO_2_ to anatase and rutile phases. Meanwhile, the intensity of the spark discharge enhances with time, meaning that prolonged oxidation times result in the formation of hydroxyapatite on the implant surface [109,110]. As such, PEO processing parameters largely affect the chemical and phase composition as well as the surface topography of the implant surface. 

### 4.4. Surface Morphology

**Figure 4:**
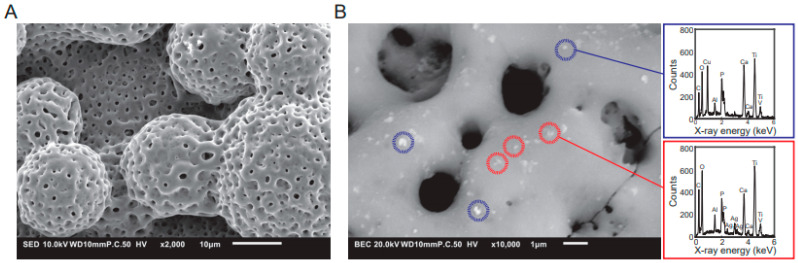
(**A**) SEM images of the typical surface morphology of titanium implants after PEO processing. (**B**) EDS analysis of the implant surface to characterize its chemical composition with spectrum of Cu (blue) and Ag (red) nanoparticles.

As PEO greatly affects the surface topography of titanium surfaces, all studies investigated the surface topography by scanning electron microscopy (SEM) and most studies reported a porous surface topography with rounded pores (Figure 4A). PEO transforms the native titanium oxide layer into a highly porous surface with interconnected porous networks, which is frequently described as a volcanic landscape with micropores that are <10 µm in diameter. In addition, flake-like morphologies [35,55,63] and needle-like structures [58] are often observed. Furthermore, the thickness and porosity of the oxide layer were shown to depend on the composition of the PEO electrolyte and PEO processing parameters [54,111]. The specifications of the surface morphology in turn were shown to greatly influence the antibacterial behavior [112] and osteogenic properties [113,114] of the implant surfaces.

### 4.5. Phase Composition by XRD

One component of the surface that plays a major role in the biological behavior is the phase composition of the implants [115]. These phases can be analyzed with X-ray diffraction (XRD). Among the included studies, 87% analyzed the phase composition. Of those, all studies analyzed Ti phases and observed bare Ti (66%), anatase (81%), and/or rutile (66%). Some studies observed both Ti and anatase, but no studies reported solely Ti and rutile. This is in line with the observation that during PEO processing, first the metastable anatase is formed, which then turns into the stable rutile [116]. While all studies that performed XRD analysis identified the TiO_2_ phases, not all studies analyzed the other phases formed by the elements incorporated from the electrolyte. Since many PEO electrolytes contain both Ca and P, 19% of the studies observed hydroxyapatite [31,35,50,55,56,57,58,64,65] and 28% contain other Ca/P phases including α-TCP [35,52,53], β-TCP [52,53], TiP_2_O_7_ [70], CaTiO_3_ [31,55,56,57,58,65], Ca_2_P_2_O_7_ [35,52], and Ca_3_(PO_4_)_2_ [31,71]. In addition, phases with Cu, Cu_2_O, and CuO [76,78], as well as ZnO [78,79,87] were observed.

These phases were shown to affect the biological response. For instance, TiO_2_ is transformed from an amorphous phase into crystalline anatase and rutile phases that were shown to produce reactive oxygen species (ROS) [117], which in turn contribute to the desired antibacterial behavior [118]. 

### 4.6. Content of the Antibacterial Elements Incorporated in the PEO Layers

The antibacterial activity of Ag, Cu, and Zn may be present on the implant surface depending on the dose [119,120,121]. Therefore, it is important to quantify the content of these elements on the implant surface after PEO biofunctionalization. This analysis is usually done either by energy-dispersive X-ray spectroscopy (EDS; Figure 4B) or X-ray photoelectron spectroscopy (XPS). Among the included studies, 80% reported the elemental composition of the surface, while 20% did not. The studies generally reported the elemental composition either in terms of atomic% or weight% and found them to correlate with the amount of Ag, Cu, and Zn dispersed in the PEO electrolyte. The amount of Ag incorporated in the implant surfaces tended to be lower (1.35 ± 1.82 wt%) than Cu (7.70 ± 10.17 wt%) and Zn (18.79 ± 12.06 wt%), reflecting the lower minimal inhibitory concentration (MIC) of Ag (0.03–8 µg/mL) as compared to Cu (256–448 µg/mL) and Zn (765 µg/mL) [122]. However, EDS does not exclusively measure the elemental composition of the surface but may penetrate deeper into the oxide layer. This is an important point, because it is not clear to what extent the species present deeper inside the oxide layer, which can be up to 10 µm in thickness, and contribute to the antibacterial properties of biofunctionalized implants [15]. The amount of active agents present on the implant surface may not be directly related to the antibacterial activity, since the form in which the element is present on the surface (i.e., ionic species, nanocrystals, or nanoparticles) affects the antibacterial properties as well [123,124].

### 4.7. Ion Release

An important antibacterial mechanism is through the release of metallic ions from the implant surface. These released ions do not only play a role in contact-killing, but also target planktonic bacteria in the implant surrounding, as this area could form a niche for bacteria [125]. Ion release was studied in 48% of the included studies and was measured from 12 h up to 56 days. Overall, the release of Ag, Cu, and Zn ions was found to be higher for the implant surfaces with a higher elemental content and a higher concentration of the active agents in the PEO electrolyte. The combination of Ag with Cu or Zn NPs on the implant surface resulted in enhanced Cu or Zn release while the Ag release was reduced in the first 24 h [19,20]. Similarly, higher concentrations of zinc acetate added to copper acetate resulted in enhanced Zn ion release while Cu ion release was reduced with higher concentrations of zinc acetate [77]. This may stem from galvanic coupling favoring the oxidation and release of one element over the other [126,127]. When studied in detail, this may allow for controlled release profiles and accompanying antibacterial effects.

Ion release results depend on the liquid in which these measurements are performed. Frequently used liquids are phosphate-buffered saline (PBS) and simulated body fluid (SBF) [128]. Ion release does not only depend on the surface content, but also on the form in which the antibacterial agent is present on the surface (i.e., as ionic species, nanoparticles, or other forms) [124]. Ideally, one could control the release of ions to not only prevent infection immediately after surgery, but also ward off late implant-associated infections [129]. However, comparing the reported ions release kinetics is difficult due to the different units, specimen designs, and measurement setups being used. In addition to the previously mentioned parameters, the surface area plays an important role in determining the concentration of the released ions, as a larger area allows for more agents to be incorporated on the surface, in turn leading to a higher release rate [31]. The reported concentrations of release ions should, therefore, be normalized with respect to the surface area of the specimens to enable direct comparison between different studies. The information regarding the surface area is generally not reported in the studies, rendering a direct comparison impossible. 

## 5. Antibacterial Properties

Surface biofunctionalization by PEO with Ag, Cu, and Zn results in antibacterial surfaces. In the following section, we will first compare the antibacterial activity of PEO biofunctionalized titanium implants bearing Ag, Cu, and Zn found by in vitro and ex vivo studies (Table 5). Then, we will discuss the factors that determine the antibacterial activity. First of all, the types of the bacterial species and strains were shown to affect the susceptibility and resistance of bacteria to antibacterial agents [130], their ability to infect host cells [131], and their pathogenicity [132]. Moreover, the type of assay, the inoculation dose, and the culture time used in the studies may affect the observed antibacterial activity. Finally, the activity against adherent and/or planktonic bacteria is discussed, as the adherence of bacteria may initiate biofilm formation, while planktonic bacteria form a source for reinfection and host cell invasion [133].

### 5.1. Comparing Antibacterial Activities of Ag, Cu, and Zn

All the included studies reported antibacterial activity. Guidelines designate a material as antibacterial when it induces a >99.9% (i.e., 3-log) reduction in the number of viable bacteria [134]. However, this is a guideline for treatment, while the required reduction in the bacterial load for the prevention of IAI is not known. In fact, 48% of the studies using Ag, 14% of the studies with Cu, 10% of the studies with Zn, and 80% of the studies that combined these metallic agents reduced the bacterial load by >99.9%. This indicates that surfaces biofunctionalized with Ag demonstrate the highest degree of antibacterial activity, while Cu and Zn were less effective, which is not surprising given the much lower MIC for Ag as compared to Cu and Zn [122]. Interestingly, combining Ag, Cu, and Zn resulted in much higher levels of antibacterial activity, while the doses of single elements can be reduced [19,20,87,88].

Studies that focused on the antibacterial mechanisms of Ag, Cu, and Zn NPs suggest that two antibacterial mechanisms play a role: ion release killing [135] and the generation of reactive oxygen species (ROS) [136]. Ions released from the implant diffuse across the bacterial cell wall and penetrate into bacteria where vital bacterial structures are targeted. Meanwhile, ROS are highly reactive and cause lysis of the bacterial cell wall. It was found that Cu showed the best antibacterial activity as a result of contact killing [137], while Ag exhibited most of its antibacterial activity through both ion release and contact killing [138]. Furthermore, the synergistic antibacterial properties of AgNPs and Zn ions were observed to stem from long-range Zn ion release and contact-killing effects from Ag through microgalvanic coupling [29,139].

**Figure 5:**
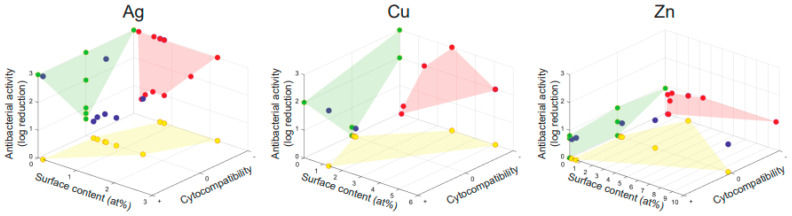
The relation between the antibacterial activity, cytocompatibility, and surface content for titanium surfaces biofunctionalized by PEO with Ag, Cu, or Zn. The reported antibacterial activity as a function of surface content and cytocompatibility is depicted by the blue dots. The green, red, and yellow projections enable a comparison between the parameters. Cytocompatibility is depicted as cytotoxicity (−), no effect (0), or enhanced cytocompatibility (+).

We plotted a 3D graph showing the correlation between antibacterial activity, cytocompatibility, and surface content of the antibacterial agent for the titanium substrates biofunctionalized by PEO with Ag, Cu, or Zn (Figure 5). Very few studies reported all of these 3 parameters. This analysis shows that Ag indeed resulted in the highest levels of antibacterial activity at lower doses compared to Cu and Zn, yet also induced cytotoxicity more frequently. However, a direct comparison between the included studies, and thereby of Ag, Cu, and Zn bearing surfaces, was hampered by a large number of variables that differ in the various studies and are addressed in the next paragraphs of this section.

### 5.2. Bacterial Species and Strains

Antibacterial results are affected by the tested bacterial species. Of the reviewed studies, 57% used *S. aureus*, 31% *E. coli*, and 12% other bacterial species, including *S. epidermidis* [63,86], *S. sanguinis* [61], *S. mutans* [57,85], *P. aeruginosa* [82], and *P. gingivalis* [64]. Given that Ag, Cu, and Zn form an alternative to antibiotics, it is important to analyze the results on antibiotic resistant bacteria, such as MRSA, which are involved in up to 32% of fracture-related infections [140,141]. MRSA was investigated in 9 studies and found to be strongly inhibited by Ag [15,31,32,50,65], Ag and Cu [19], Ag and Zn [20], Cu and Zn [88] bearing surfaces, while one study that included Zn surfaces did not observe any inhibition [86]. Thukkaram et al. observed that the antibacterial effect of Ag containing surfaces against MRSA was lower compared to *S. aureus* and *E. coli*, although with increasing doses of Ag, all bacterial species were targeted equally [65]. Furthermore, testing on multiple species was performed in 19% of the included studies. No studies tested multiple species in a single experiment (i.e., co-culture of multiple species), which would be of interest given that 10–20% of IAI are induced by polymicrobial infections [142,143]. 

We can, thus, conclude that most studies investigated antibacterial behavior against *S. aureus*. This bacterial species causes 20–46% of IAI [144,145,146]. Other gram-positive species, such as *Streptococci* caused up to 10% and *Enterococci* 3–7% of cases [147]. *Enterococci* have not been tested in studies with PEO-treated surfaces bearing Ag, Cu, or Zn. Gram-negative bacteria, such as *Pseudomonas aeruginosa* and *Enterobacteriaceae* induce 6–17% of IAI [143,148]. Given the relatively low rate of IAI induced by *Enterobacteriaceae*, it is surprising that 31% of the studies investigated the effects of the implant surfaces on *E. coli*. While some studies that analyzed both *S. aureus* and *E. coli* reported a stronger antibacterial effect against *E. coli* as compared to *S. aureus* [49,51,55,65,83,98], others reported a similar antibacterial effect for both species [35,37,70,74,84,88]. Interestingly, up to 42% of IAI in patients were caused by culture-negative (i.e., undefined) bacteria [149,150] and therefore warrant an antibacterial agent effective against a wide antimicrobial spectrum. 

Among bacterial species, different levels of sensitivity to antibacterial agents have been reported [151], including against Ag and Cu [152]. To what extent the differences between strains plays a role depends on the bacterial species. The differences between strains in terms of their MIC/MBC values was found to be negligible for *S. aureus,* but were quite large in the case of *E. coli* strains [153]. It is, therefore, important that the bacterial strain is properly reported, which was done only in 79% of the included studies. Only one study, conducted by Leśniak-Ziółkowska et al., compared different strains within a bacterial species, namely *S. aureus* (ATCC 25,923 and clinical MRSA 1030) and *S. epidermidis* (ATCC 700,296 and clinical 15560) [86]. No strain-dependent differences were observed after 4 h using a bacterial adhesion test.

### 5.3. Source of Antibacterial Agent

Antibacterial behavior depends not exclusively on the antibacterial agent, but also on the form in which Ag, Cu, and Zn are added to the PEO electrolyte and are subsequently incorporated onto the titanium implant surface [124]. Ag, Cu, and Zn elements are either completely dissolved in the electrolyte or are added in the form of NPs that form a suspension. The former will end-up in the form of chemical compounds present all over the surface, while the latter (NPs) are spread over the surface. NPs may form a reservoir from which ions are released, thereby ensuring prolonged antibacterial activity [154]. In addition, the shape of the NPs determines the antibacterial activity as the surface-to-volume ratio affects the ion release and, thus, the efficacy of the surface biofunctionalization process [155]. Ionic forms only induce antibacterial activity through the action of ions, while NPs also produce reactive oxygen species and induce contact-killing [156]. Among the included studies, 33% used NPs, 64% employed ionic species, and only a study by Zhang et al., combined ions and NPs [87]. This study combined Ag NPs with Zn acetate, which resulted in much higher release of Zn ions compared to Ag ions. Furthermore, the antibacterial activity was assessed against both adherent and planktonic *S. aureus* after 24 h. The developed surface demonstrated significant antibacterial behavior with increasing concentrations of Ag and Zn leading to further reduction of viable bacteria. The authors reasoned that the antibacterial activity stems from ROS generation by both Ag and Zn as well as Ag^+^ release. Moreover, both Ag and Zn ion concentrations remained below cytotoxicity levels and thus stressed the utility of combining these elements. Studies that investigate the differences in the antibacterial properties induced by NP and ionic forms are lacking.

### 5.4. Analysis Method

Antibacterial properties can be investigated by different assays. Properties often investigated are the antibacterial leaching activity, the killing of adherent bacteria, and the prevention of biofilm formation. Although most of the included studies used only one antibacterial assay (53%), the use of several assays is required for the assessment of the various types of antibacterial properties [157]. Therefore, 32, 8, and 8% of the included studies used 2, 3, and 4 assays, respectively. To determine the leaching effects of the antibacterial ions released from the PEO surfaces, a zone of inhibition assay or a Kirby-Bauer assay is often used. The number of bacteria can be quantified either through a direct CFU count, by spread plate analysis, or by staining the live cells using a fluorescent dye. A few studies referred to ISO [51,59] and ASTM [37,76,78] standards. With SEM, adherent bacteria and/or biofilm formation can be visualized in a non-quantitative manner. A wide variety in the type of assays used in the studies was found, with spread plate analysis (33%), SEM (24%), and viability fluorescence imaging (12%) being the most frequently applied assays.

In addition to in vitro assays, ex vivo models were explored, in which infected implants biofunctionalized with Ag and Cu, Zn, or Sr are inserted into a murine femur [19,20,31,50]. Subsequently, the number of CFU present are quantified (e.g., after 24 h). Although this ex vivo model does not allow to assess the effects of the implants on the immune system or bony ingrowth, some of the other in vivo effects such as those of the extracellular matrix and bone tissue [158] can be captured to some extent. Indeed, the gene expression profile of osteocytes was found to be similar between an ex vivo bone infection model and tissue samples from IAI patients [159]. Thus far, no study has tested the antibacterial activity of titanium implants biofunctionalized by PEO with Ag, Cu, and Zn in vivo.

### 5.5. Duration and Inoculum of Antibacterial Assay

Over two thirds of IAIs are initiated during surgery [160]. A rapid antibacterial response to prevent the adherence of the bacteria that enter the human body peri-operatively is, therefore, desired. Almost all of the included studies (94%) tested the antibacterial properties within 24 h and 10% even within 2 h. However, IAI can also be initiated long after surgery, stemming from hematogenous origins. Prolonged antibacterial activity is, thus, desirable too [72,85,161]. Zhang et al., reported on the antibacterial activity of Cu-containing surfaces for longer periods of time [72]. It was observed that the number of viable adherent bacteria was significantly reduced on surfaces containing 0.67–1.98 wt% Cu up to 96 h. However, this was one of the few studies aiming to assess long-term antibacterial behavior, since prolonged in vitro culture of bacteria is challenging. Research into late IAI is, therefore, primarily performed in vivo [162,163].

The inoculum used in the antibacterial assays is another factor determining the antibacterial behavior of PEO-biofunctionalized implants. The exact number of bacteria required for IAI is unknown, but it was shown that the presence of a foreign body can reduce the infection dose by 6 orders of magnitude [164] due to a hampered immune response [165]. The inoculum used in the included studies varied widely between 250 [53] and 10^9^ CFU/mL [58,85], and was not reported in two studies. Currently, most inocula are presented per volume or as a measure of optical density. However, the surface area of the implant is also of importance, as more area with more incorporated antibacterial agent is likely to have a greater antibacterial effect. Therefore, presenting the inoculum per volume per surface area would support comparative analyses of different studies.

### 5.6. Planktonic vs. Adherent Bacteria

As both planktonic and adherent bacteria play an important role in IAIs, antibacterial implants should target both types of bacteria. Planktonic bacteria are present in the fluid and tissue surrounding the implant and have shown to be a reservoir for late-stage reinfections [125]. Once the bacteria adhere to the implant, bacteria should be targeted in order to prevent biofilm formation as this would induce bacterial resistance to antibiotic treatment [166]. In this respect, 42% of the included studies investigated antibacterial activity against adherent, 35% against planktonic, and 23% against both planktonic and adherent bacteria. Targeting both planktonic and adherent bacteria should, therefore, be emphasized more in future studies.

## 6. Biocompatibility

In addition to antibacterial properties, PEO-biofunctionalized implant surfaces should not induce cytotoxicity, and ideally even enhance cell response and bony ingrowth. The compatibility of the implants with mammalian cells is, therefore, an important topic that needs to be thoroughly investigated for any such implant. Several of the included studies report the results of such in vitro cytocompatibility experiments, which are affected by the type of the assay, cell type, and cell source (Supplementary Table S1). 

### 6.1. Cytocompatibility of Ag, Cu, and Zn Surfaces

Cytocompatibility was investigated in 71% of studies. In those studies, Ag induced cytotoxicity in 13% of the studies, while 10% of the studies investigating Cu and 0% of those employing Zn reported cytotoxic effects. None of the studies combining Ag, Cu, and Zn reported cytotoxicity. Cell response of the implants was improved in 7% of the studies using Ag, 50% of the studies focused on Cu, and 33% of the studies with Zn, as well as for 50% of the studies in which two or more antibacterial agents were combined. The control group often consists of PEO biofunctionalized surfaces without antibacterial elements. Cytotoxicity is, therefore, not considered a major concern by the vast majority of the included studies. Indeed, Cu and to somewhat lesser extent Zn were shown to improve the cytocompatibility of PEO-treated implants.

### 6.2. Type of Assay

Several processes that occur in bone regeneration were investigated in vitro. Cells need to attach to the implant surface [167], spread [168], stay viable [169], proliferate, differentiate towards the osteogenic lineage [170], and eventually form an extracellular matrix [171]. Indicators for the bone regeneration process include cell morphology [172], expression of osteogenic markers [173], metabolic activity [174], and the production of specific proteins [175]. The parameters studied the most in the included studies were viability and proliferation (analyzed in 56% of the included studies), followed by adhesion and attachment (36%), differentiation (25%), cell spreading (22%), matrix calcification and mineralization (11%), metabolic activity (8%), gene expression (8%), morphology (3%), cell seeding (3%), and other assays (6%) including protein production, mitochondrial functioning, and cytokine production.

### 6.3. Cell Type

The cellular response was shown to differ in in vitro experiments between different cell types [176,177]. In the reviewed studies, pre-osteoblasts (32%), osteosarcoma cells (22%), fibroblasts (20%), MSCs (17%) and SV-HFO, macrophages, adipose stem cells, and endothelial cells (each in 1 study) were used. Pre-osteoblasts and MSCs are the main cells responsible for bone formation [178,179]. Osteosarcoma and SV-HFO cells [180] are immortalized cells stemming from the osteogenic lineage. However, osteosarcoma was shown to stem from defective differentiation [181]. Since these titanium implants will be used in bone tissue, it was surprising that 29% of the studies did not analyze the effects of the implants on bone-forming cells. Other cell types may support bone formation through indirect pathways. Endothelial cells play a role in angiogenesis, which plays a major role in bone regeneration as blood vessels carry nutrients and oxygen and facilitate the transport of immune cells to the regenerating bone tissue [182]. Meanwhile, macrophages form an important part of the immune response against IAI. Any potential toxicity of the synthesized implants against this cell type is of concern, as it may hamper the clearance of infections [16,183]. Finally, fibroblasts were shown to regulate osteoblast activity through tight junction interactions [184].

### 6.4. Cell Source

About 22% of the included studies used primary cells, whereas 78% utilized cell lines. Primary cells are more representative of the clinical situation, as they have been isolated from donors. However, their variability is high. Cells from multiple donors, therefore, need to be tested [185]. Cell lines, on the other hand, are homogenous and stable, while exhibiting little variability. However, their immortalized nature makes them differ from the clinical situation [186]. Furthermore, the source of animal species from which the cells were derived differed greatly between the included studies, with 56% using murine cells, 34% human cells, and 10% rat cells. The osteogenic differentiation capacity of stem cells is known to differ between human, mice, and rat MSCs [187,188]. These differences in animal species make it difficult (if not impossible) to directly compare the cytocompatibility results reported in the different studies. 

## 7. Discussion

In order to prevent IAI, the biofunctionalization of titanium implants by PEO using Ag, Cu, and Zn as the active agents has gained significant momentum in the last decade. Therefore, we systematically reviewed the progress made on those implants and summarized the various types of properties measured for such types of PEO-biofunctionalized implants.

### 7.1. Antibacterial Results

From the results of this study, it can be concluded that Ag is the most potent antibacterial agent followed by Cu and Zn. It is important to stress that different studies utilized different experimental protocols to determine the antibacterial properties of PEO-biofunctionalized implants. It was shown that titanium surfaces bearing Ag, Cu, and Zn can kill bacteria through antibacterial leaching activity, contact killing, and the formation of ROS [156,189]. These properties cannot be assessed in a single assay. The use of multiple assays is, therefore, warranted to support the claim of antibacterial activity [157]. Finally, it is important to make sure that the assays assess infection prevention rather than infection treatment.

Furthermore, the bacterial species and strains used were found to affect the level of antibacterial activity. For instance, surfaces demonstrating antibacterial activity against *E. coli* may not do the same against *S. aureus* [51,55,98]. Most studies investigated the antibacterial activity of the implants against *S. aureus* or *E. coli*. While a large proportion of IAI was induced by *S. aureus*, only a small proportion of infections were caused by *E. coli* [144,147]. The rationale for choosing *E. coli* was, thus, primarily methodological convenience rather than clinical prevalence. Meanwhile, *S. epidermidis* or polymicrobial infections were rarely studied, even though they cause a significant proportion of IAI [142,143,144]. Moreover, the antibacterial behavior of PEO-biofunctionalized implants should be assessed in environments co-habited by multiple bacterial species, as this was shown to influence the resistance profiles of bacteria [130].

The antibacterial experiments aimed to mimic the clinical situation as closely as possible. In this respect, both adherent and planktonic bacteria should be warded off, as adherent bacteria can form biofilms [133], while planktonic bacteria may infect the peri-implant tissue and form a reservoir for late-stage reinfection [125]. Furthermore, an antibacterial implant should prevent infections that occur immediately after surgery, as that is the point where most IAI occur [160], as well as late-stage infections from hematogenous origins [161]. At the moment, the focus primarily lies on preventing early-stage infections. Ultimately, Ag, Cu, and Zn may form an alternative to antibiotics, as bacteria are developing ever-growing degrees of antibiotics resistance [144,190]. As such, the development of resistance against Ag, Cu, and Zn and combination thereof is worthwhile to investigate given that resistance against Ag, Cu, and Zn was reported in vitro [191,192,193] and in patients [194].

The observed antibacterial activity depends on a wide variety of factors described in this review, including the titanium substrate, composition of the PEO electrolyte, and PEO processing parameters that in turn affect the surface morphology, phase composition, surface content of the incorporated antibacterial agent, and ion release profile. These parameters determine the antibacterial properties and biocompatibility of the implants. The measured antibacterial properties are highly dependent on the bacterial species and strains used, experimental techniques, the duration of the assays, bacterial inoculum, and the type of bacteria against which the implant performance is measured (i.e., planktonic and/or adherent). As for biocompatibility, the type of the assays, cell type, and cell source could all influence the final read-outs. These factors varied between the studies included in this review and make a one-to-one comparison between the different studies challenging.

The antibacterial activity is dependent on the dose of Ag, Cu, and Zn present on the surface of the titanium implants [119,120,121]. It is, therefore, essential to determine the amount of these elements present on the surface. In addition, the Ag, Cu, and Zn ions released from the implant surface are responsible for a significant part of the antibacterial activity, which is why it is important to measure the concentration of the ions released from the implant surface. From the results, it is clear that the surfaces bearing Ag had much lower elemental content and ion release as compared to those bearing Cu and Zn, which was expected due to the lower MIC of Ag as compared to Cu and Zn [122]. Both the surface content and ion release were also dependent on the surface area, as a larger surface area allows for the incorporation of a greater amount of elements and, thus, increased ion release [31]. Therefore, describing these properties relative to the surface area may aid in a comparison between the results of different studies.

### 7.2. Biocompatibility

Most of the included studied found cytotoxicity to be a minor concern, with Ag inducing cytotoxicity in 13% of the studies. It was striking that 29% of the included studies did not investigate the effects of the implants on bone-forming cells, even though the implants are intended for bone tissue. In addition, cytotoxicity against other cell types, such as endothelial cells and immune cells is of interest, as these cells contribute to bone regeneration as well [195,196]. Furthermore, the use of cell lines vs. donor cells and different mammalian species complicates the comparisons between different studies [197]. Moreover, biocompatibility needs to be investigated both in vitro and in vivo, as the results of in vitro and in vivo experiments are known to differ, for instance, in the case of Ag-bearing surfaces [16].

Another way to enhance the cytocompatibility of PEO-biofunctionalized implants is by combining two or more antibacterial metals (i.e., Ag, Cu, and Zn), as synergic effects between various such agents are reported to exist [19,20] and could be used to reduce the concentration of Ag [126,198]. In addition, combining these elements with other osteogenic elements, such as Sr [50] may enhance their antibacterial and biocompatible properties. Finally, the combination of multiple antibacterial elements significantly reduces the risk of the development of bacterial resistance, thereby ensuring that the prolonged use of these elements will remain possible [199].

PEO is frequently applied in combination with other surface treatments, such as hydrothermal treatment [200] and physical vapor deposition [201] to alter the chemical and phase composition of the surface. This may result in improved antibacterial behavior [98]. Furthermore, hydrothermal treatment has resulted in the enhanced formation of hydroxyapatite crystals, yet may reduce corrosion resistance too [202]. A major disadvantage of these additional surface treatments is that they make the entire process lengthier and more complex, thus making it more difficult to upscale the production of clinically sized implants.

### 7.3. Towards Clinically Relevant Implants

A decade of PEO biofunctionalization of titanium implants with Ag, Cu, and Zn confirmed the great potential of this method as an effective, fast, and scalable process. At the moment, however, the research on antibacterial PEO-biofunctionalized titanium implants is still far away from clinical application, as the research was primarily conducted in vitro with few studies also exploring ex vivo models [20,50]. Furthermore, PEO was shown to enhance the osteogenic capacity of titanium implants in vivo [38,39,203], including surfaces bearing Zn [204]. However, these studies did not analyze the antibacterial properties of such implants, which should be evaluated using bone infection models [205]. In this respect, a major limitation of the state-of-the-art techniques is their limited relevance for the assessment of the preventive potential of antibacterial implants (as opposed to their treatment potential). However, studying prevention requires a much larger sample size, as it is associated with lower bacterial loads, meaning that infections are less likely to occur. This lower risk of infection has major ethical and financial implications. In addition, future implants will most likely be fabricated by AM and as such be highly porous. Not only is the risk of infection of such volume-porous implants higher, their IAI treatment is also highly challenging due to their usually high degree of bony ingrowth that may cause significant bone loss during their removal. The development of antibacterial surface treatments for such types of implants is, thus, highly relevant. In fact, the additional surface area of such implants may be exploited to enhance the bioactivity of PEO biofunctionalized implants [31]. 

## 8. Conclusions

In order to combat IAI, the biofunctionalization of titanium implants by Ag, Cu, and Zn has gained significant momentum in recent years and resulted in the synthesis of potent antibacterial and biocompatible surfaces. Implant biofunctionalized with Ag, Cu, and Zn demonstrated significant antibacterial behavior against a wide bacterial spectrum, including antibiotic-resistant bacterial strains. However, the antibacterial properties of these implants were primarily investigated in vitro and occasionally ex vivo. Furthermore, many studies do not reach sufficiently high antibacterial levels, as indicated by international guidelines. Moreover, the biofunctionalization of volume-porous AM implants has not been investigated extensively. Finally, combining Ag, Cu, and Zn on the surface of titanium implants was shown to result in potent antibacterial surfaces with reduced cytotoxicity. In order to take the PEO biofunctionalization of titanium implants by Ag, Cu, and Zn to clinical settings, in vivo studies should be conducted using relevant infection models for both solid and volume-porous bone implants.

## Figures and Tables

**Table 1:** The methodological details of the included studies in which Ag was used as the antibacterial agent.

			PEO Processing Parameters							
Substrate	# of Exp Groups with Ag	Electrolyte	Voltage (V)	Current Density (A/dm^2^)	Time (min)	Surface Topography	Phase Composition	Surface Content of Ag	Cumulative Ag Ion Release (ppb)	Ref.
Ti6Al7Nb	2	0.02 M CA, 0.15 M Ca-GP, and (0.3 and 3.0) g/L Ag NPs	-	20	5	Porous structures (<5 µm)	-	-	12—day 7 89—day 7	[15]
Ti6Al4V	2	0.15 M CA, 0.02 M Ca-GP, and 3.0 g/L Ag NPs	-	20	5	Micro- and nano-porous structures with Ag NPs of 7–25 nm	Ti, anatase, rutile, HA, CaTiO_3_, and Ca_3_(PO_4_)_2_	-	138—day 28 600—day 28	[31]
Ti6Al7Nb	1	0.15 M CA, 0.02 M Ca-GP, and 3.0 g/L Ag NPs	-	20	5	Porous structures (<3 µm) with Ag NPs of 37 nm	Ti, anatase, and rutile	0.03 wt%	-	[32]
CP-Ti	3	0.4 M CA, 0.04 M β-GP, and (0.00003, 0.00006 and 0.004 M) AgNO_3_	380–420	-	180	Irregular and rough morphology with spherical particles and flakes	Rutile, α-TCP, β-Ca_2_P_2_O_7_, and HA	<0.1 wt% <0.1 wt% 0.21–0.45 wt%	-	[35]
CP-Ti	1	0.15 M CA, 0.05 M NaH_2_PO_4_, 0.25 mM AgNO_3_	280–320	-	6	Porous surface with 1.5 µm pore size and 8.5% pore density	Anatase, rutile	0.13 at%	48—day 18	[48]
CP-Ti	3	0.4 g/L NaOH, 4.0 g/L NaH_2_PO_4_, and 0.1–1.0 g/L Ag NPs	400	-	5	Homogenous porous surface layer	Ti, anatase, rutile	1.5 at% 3.5 at% 5.8 at%	40—day 7 200—day 7 240—day 7	[49]
Ti6Al4V	2	0.15 M CA, 0.02 M Ca-GP, 0.3 M SrA, and 3.0 g/L Ag NPs	-	20	5	Uniform coverage with a micro-/nanopores. Addition of SrA resulted in smaller pore size.	Ti, anatase, rutile, HA, SrTiO_3_, Sr_2_Ca(PO_4_)_2_	-	1500—day 28 1800—day 28	[50]
CP-Ti	3	100 mM Ca-GP, 150 mM CA, 0,5, and 10 mM AgNO_3_	-	2.51	10	Porous oxide layer for 0 and 5 mM Ag, non-porous surface for 10 mM Ag	Anatase, α-Ti	0.5 at% 1.5 at% 3.0 at%	300—day 28 3000—day 28 10^4^—day 28	[51]
CP-Ti, Ti-40Nb	2	Na_2_HPO_4_, NaOH, β-Ca_3_(PO_4_)_2_, and 0.3—1 g/L AgNO_3_	200–450	-	5–10	Uniformly distributed β-TCP particles over a porous surface with 0–8 µm pore sizes	Anatase, α-TCP, β-TCP	0.2 at% 0.8 at%	-	[52]
CP-Ti	4	Na_2_HPO_4_, NaOH, β-Ca_3_(PO_4_)_2_, and 1 g/L AgNO_3_	200–450	-	5–10	Uniformly distributed β-TCP particles over a porous surface with 0–8 µm pore sizes	Anatase, α-TCP, β-TCP	0.3 at% 0.5 at% 0.8 at%	-	[53]
CP-Ti	3	0.1 M CA, 0.06 M NaH_2_P, and 0.01—0.05 M Ag_2_O NPs	-	10	10	Porous structure with typical micro-sized pores	Anatase, rutile	1.6 wt% 3.1 wt% 5.8 wt%	2000—day 28 4000—day 28 10^4^—day 28	[54]
CP-Ti	1	CA, Na_2_HPO_4_, and 0.0025 M Ag-A	380	-	5	Flake-like morphology with regional Ag particles of <200 nm	Ti, anatase, rutile, HA, and CaTiO3	4.6 wt%	-	[55]
CP-Ti	3	20.5 g/L CA, 7.2 G/L Na_2_HPO_4_, and (0.0005, 0.001, and 0.002) M Ag-A	400	-	5	Micro-porous structures with Ag NPs surrounding micro-pores	Ti, anatase, rutile, HA, and CaTiO3	1.14 wt%	-	[56]
Ti6Al4V	1	20.5 g/L CA, 7.2 g/L Na_2_HPO_4_, and 0.001 M Ag-A	400	-	5	Micro-porous structures with Ag NPs of <100 nm surrounding micro-pores	Ti, anatase, rutile, HA, and CaTiO3	0.7 wt%	1500—day 14	[57]
Ti6Al4V	2	CA, β-GP and (0.1 and 0.4) g/L AgNO_3_	400	-	5	Granular and needle-like morphology with Ag NPs of 20–30 nm	Ti, anatase, rutile, HA, and CaTiO_3_	0.6 wt% 2.1 wt%	2500—day 14 8000—day 14	[58]
Ti-29Nb-13Ta-4.6Zr	2	0.15 M CA, 0.1 M Ca-GP, and (0.0005 and 0.0025) M AgNO_3_	-	2.51	10	Porous structures (<10 µm)	-	0.01 wt% 0.01 wt%	-	[59]
CP-Ti	3	0.1 M KOH, 0.015 M K_4_P_2_O_7_, and (0.1, 0.3 and 0.5) g/L Ag NPs	-	10	5	Micro-porous structures with Ag NPs of <20 nm (3–7.5 µm)	-	0.53 at% 0.69 at% 0.80 at%	12.2—day 1 22.7—day 1 28.8—day 1	[60]
CP-Ti	1	0.3 M CA, 0.02 M GP, and 0.62 g/L Ag NPs	290	-	10	Porous structures with volcano top-like micro-pores	Ti, anatase, and rutile	1.07 at%	-	[61]
CP-Ti	1	0.3 M CA, 0.02 M GP, and 0.62 g/L Ag NPs	290	-	10	Porous structures with Ag NPs of <100 nm	Ti, anatase, and rutile	-	-	[62]
Ti6Al4V	1	Pure water and AgPURE^TM^ W10 nanosilver suspension	-	20	0.5	Flake-like morphology with Ag particles of <200 nm	-	3.6 at%	-	[63]
Ti6Al4V	2	0.2 M CA, 0.02 M β-GP, and (0.005 and 0.05) g/L Ag NPs	387 ± 3 385 ± 2	8	3	Porous structures with volcano top-like micro-pores (<3 µm)	Ti, rutile, and HA	<0.1 wt% <0.1 wt%	-	[64]
CP-Ti	3	2.0 g/L NaH_2_PO_4_·2H_2_O, 5.0 g/L CA, and 0.1, 0.5, and 0.8 g/L Ag-A	500	-	5	Porous structures uniformly covering surface	Ti, anatase, rutile, HA, CaTiO_3_	0.8 at% 1.5 at% 2.2 at%	264—day 7 813—day 7 1110—day 7	[65]
CP-Ti	2	NTA, Ca(OH)_2_, and 180 mg/L Ag NPs	250–300	-	5	Rough, thick oxide layer with a highly porous structure	-	0.3 wt% 0.7 wt%	-	[66]

Ag-A: silver acetate, CA: calcium acetate, Ca-GP: calcium glycerophosphate, GP: glycerophosphate, HA: hydroxyapatite, KOH: potassium hydroxide, NPs: nanoparticles, NTA: nitrilotriacetic acid, SrA: strontium acetate, TCP: tricalcium phosphate.

**Table 2:** The methodological details of the included studies in which Cu was used as the antibacterial agent.

			PEO Processing Parameters							
Substrate	# of Exp Groups with Cu	Electrolyte	Voltage (V)	Current Density (A/dm^2^)	Time (min)	Surface Topography	Phase Composition	Surface Content of Cu	Cumulative Cu Ion Release (ppb)	Ref.
CP-Ti	1	0.1 M CA, 0.05 M GP, and 0.05 M Cu(OAc)_2_	-	16.5	4	Micro-porous or crater structures (3–5 µm) with nano-grains of 30–50 nm	Ti and anatase	1.4 ± 0.08 wt%	-	[36]
CP-Ti, Ti-40Nb	2	H_3_PO_4_, 50–75 g/L CaCO_3_, 40–60 g/L Cu-substituted HA	200–450	-	5–10	Uniformly distributed β-TCP particles over a porous coating surface with 0–8 µm pore sizes.	Anatase, β-TCP, α-TCP, Ca_2_P_2_O_7_	0.1 at% 0.2 at%	-	[52]
CP-Ti	1	0.02 M C_12_H_22_CaO_14_, 0.01 M (NaPO_3_)_6_, 0.02 M C_12_H_22_CuO_14_	NR	NR	6	Porous surface with irregularly shaped and sized pores	-	-	-	[67]
CP-Ti	2	0.1 M CA, 0.06 M NaH_2_P, 5–10 g/L Na_2_Cu-EDTA	-	10	10	Highly porous area with micro-sized pores and a rough less porous area	-	2.3 wt% 4.2 wt%	3.3/cm^2^—day 8 8.1/cm^2^— day 8	[68]
CP-Ti	3	H_3_PO_4_, 300–600 g/L Cu(NO_3_)_2_∙H_2_O	450	-	5	With increasing Cu-salt levels sharpening of pores	Ti, anatase	0.54 at% 0.55 at% 0.72 at%	-	[69]
Ti6Al4V	2	11 g/L KOH, 10 g/L EDTA-CuNa_2_, 5 or 15 g/L phytic acid	-	10	3	Uniformly distributed three-dimensional porous structure	Anatase, rutile, and TiP_2_O_7_	1.01 wt% 1.92 wt%	192—day 8 197—day 8	[70]
CP-Ti	1	0.2 M CA monohydrate, 0.02 M NaH_2_PO_4_, 0.01 M CuA monohydrate	-	3.25	5	Volcanic uniform porous morphology with 1–5 µm pores	Ti, rutile, anatase, Ca_3_(PO_4_)_2_	5.05 at%	32.8—day 14	[71]
CP-Ti	4	0.2 M CA, 0.02 M β-GP, and (0.00125, 0.0025, 0.00375, and 0.005) M Cu(OAc)_2_	450	-	1.5	Micro-porous structures (1–4 µm)	Ti, anatase, and rutile	0.67 wt% 1.17 wt% 1.51 wt% 1.93 wt%	6.75—day 21 - - 60.2—day 21	[72]
CP-Ti	2	0.1 M Na_2_, 0.25 M NaOH, 0.1 M CA, 0.02 M Na_2_SiO_3_, and (0.0002 and 0.002) M CuSO_4_	250	-	5	Macro-pores or crater structures (>100 µm) with nano-grains	-	-	411.3—day 2 27.0—day 2	[73]
CP-Ti	1	15 g/L NaH_2_PO_4_, 2 g/L NaOH, and 3.0 g/L Cu NPs	-	20	5	Porous structures (<5 µm) with Cu NPs of <60 nm	Ti, anatase, and rutile	-	-	[74]
CP-Ti	2	15 g∙L-1 NaH_2_PO_4_, 2 g/L NaOH, and (0.3 and 3.0) g/L Cu NPs	470 ± 3 465 ± 3	20	5	Micro-porous structures (1–5 µm)	Ti, anatase	1.30 at% 2.76 at%	0.117—day 1 0.135—day 1	[75]
Ti6Al4V	3	Phosphate electrolyte with (2,6 and 10) g/L Cu_2_O NPs	450	-	15	Micro-porous structures (<30 µm) with Cu_2_O NPs of 20–30 nm	Ti, anatase, rutile, Cu, Cu_2_O, and CuO	16.0 wt% 23.2 wt% 24.5 wt%	-	[76]
CP-Ti	1	0.002 M CA, 0.02 M β-GP, and 0.0013 M Cu(OAc)_2_	480	-	2	Micro-porous structures (1–4 µm)	Ti, anatase, and rutile	0.77 wt%	4.5—day 7	[77]
Ti6Al4V	1	50 g/L Na_2_SiO_3_ and 4 g/L Cu_2_O NPs	350	-	15	Porous structures (<3 µm) with Cu_2_O NPs of 20–50 nm	Ti, anatase, rutile, Cu, Cu_2_O, and CuO	27.27 wt%	-	[78]

CA: calcium acetate, Ca-GP: calcium glycerophosphate, CuA: copper acetate, GP: glycerophosphate, HA: hydroxyapatite, KOH: potassium hydroxide, NPs: nanoparticles, NR: not reported, TCP: tricalcium phosphate.

**Table 3:** The methodological details of the included studies in which Zn was used as the antibacterial agent.

			PEO Processing Parameters							
Substrate	# of Exp Groups with Zn	Electrolyte	Voltage (V)	Current Density (A/dm^2^)	Time (min)	Surface Topography	Phase Composition	Surface Content of Zn	Cumulative Zn Ion Release (ppb)	Ref.
CP-Ti	3	20 g/L Na_3_PO_4_, 4 g/L NaOH, and (5, 10, and 15) g/L NPs	301 304 310	1000	7	Porous structures with ZnO NPs of 25 nm (<1.51–0.98 µm)	Ti, anatase, and rutile	20 wt% 25 wt% 35 wt%	-	[37]
CP-Ti, Ti-40Nb	2	H3PO4, 50–75 g/L CaCO_3_, 40–60 g/L Zn-substituted HA	200–450	-	5–10	Uniformly distributed β-TCP particles over a porous coating surface with 0–8 µm pore sizes	Anatase, β-TCP, α-TCP, Ca_2_P_2_O_7_	0.28 at% 0.4 at%	-	[52]
Ti6Al4V	1	50 g/L Na_2_SiO_3_ and 4 g/L ZnO NPs	350	-	15	Porous structures (<3 µm) with ZnO NPs of 20–50 nm	Ti, anatase, rutile, and ZnO	35.54 wt%	-	[78]
CP-Ti	2	0.1 M CA, 0.06 M NaH_2_P, 0.02 M Na_2_Zn-EDTA, or 0.02 M ZnO NPs	-	10	10	Porous surface at micrometer scale	Anatase, rutile, ZnO	-	-	[79]
CP-Ti	3	0.15 M CA, 0.1 M Ca-GP, 0.5–2.5 mM ZnCl_2_	-	2.51	10	Continuous porous surface with circular pores of 5.3 µm in size	α-Ti, anatase	3.3 at%	250—day 7	[80]
CP-Ti	1	15 g EDTA-_2_Na, 8.8 g Ca(CH_3_COO)_2_·H_2_O, 6.3 g Ca(H_2_PO_4_)·H_2_O, 7.1 g Na_2_SiO_3_·9H_2_O, 5 g NaOH, 6 mL H_2_O_2_, 8.5 g Zn(CH_3_COO)_2_ in 1 L	350–500	-	7	Porous and rough surface with 1–3 µm pore sizes increasing voltages resulting in decreasing pore density and increased pore sizes	Ti, anatase, rutile	2 at%	250—day 15	[81]
CP-Ti	1	0.15 M CA, 0.15 M Ca-GP, and 0.02 M ZnA	350	-	1	Porous structures with volcano-shaped structures	Ti, anatase, and rutile	9.7 at%	300—day 1 <1000—day 28	[82]
CP-Ti	3	0.1 M CA, 0.05 GP, and (0.02, 0.04, and 0.06) M ZnA	-	16.5	4	Porous (<5 µm) with nano-grains of 20–100 nm	Ti, anatase, and rutile	4.6 ± 0.7 wt% 7.1 ± 0.6 wt% 9.3 ± 0.8 wt%	1180—day 14 2235—day 14 3620—day 14	[83]
CP-Ti	1	0.02 M CA, 0.15 M Ca-GP, and 0.06 M ZnA	-	30	5	Porous structures (<5 µm)	Ti, anatase, and rutile	8.7 at%	-	[84]
CP-Ti	3	0.1 M CA, 0.025 M Na_5_P_3_O_10_, and (0.01, 0.03, and 0.05) M ZnA	380	-	20	Micro-porous structures	-	0.199 at% 0.574 at% 1.995 at%	-	[85]
Ti-15Mo	3	0.1 M Ca(H_2_PO_2_)_2_, 10 g/L ZnO, or 25 g/L Zn_3_(PO_4_)_2_ or 10 g/L Ca_3_(PO_4_)_2_ and 10 g/L Zn_3_(PO_4_)_2_ particles	300	15	5	Porous oxide layer with micropores	-	1.5 at% 1.1 at% 0.2 at%	115—week 16 64—week 16 60—week 16	[86]

CA: calcium acetate, Ca-GP: calcium glycerophosphate, GP: glycerophosphate, HA: hydroxyapatite, KOH: potassium hydroxide, NPs: nanoparticles, NR: not reported, TCP: tricalcium phosphate, ZnA: zinc acetate.

**Table 4:** The methodological details of the included studies in which multiple antibacterial agents were used.

			PEO Processing Parameters							
Substrate	# of Exp groups	Electrolyte	Voltage (V)	Current Density (A/dm^2^)	Time (min)	Surface Topography	Phase Composition	Surface Content of Zn	Cumulative Ion Release (ppb)	Ref.
Ag and Cu										
Ti6Al4V	6	0.15 M CA, 0.02 M Ca-GP, 0.75–3.0 g/L Ag, and/or Cu NPs in ratios 0–100%	-	20	5	Homogeneous porous surface with circular pores. Ag and/or Cu NPs scattered on surface.	-	-	Day 28: 1491 (Ag)/- 1906 (Ag)/- 1573 (Ag)/1527 (Cu) 1425 (Ag)/1392 (Cu) 1291 (Ag)/1225 (Cu) -/1981 (Cu)	[19]
Ag and Zn										
Ti6Al4V	6	0.15 M CA, 0.02 M Ca-GP, 0.75–3.0 g/L Ag, and/or Zn NPs in ratios 0–100%	-	20	5	Homogeneous porous surface with circular pores. Ag and/or Zn NPs scattered on surface.	-	-	Day 28: 1491 (Ag)/- 1906 (Ag)/- 1573 (Ag)/1467 (Zn) 1682 (Ag)/1697 (Zn) 1749 (Ag)/1678 (Zn) -/2281 (Zn)	[20]
CP-Ti	3	0.1 M CA, 0.02 M β-GP, 0.25 g∙L-1 SDBS, 0.1 M ZnA, and 6 g/L Ag NPs	390	-	0.5 1.5 2	Micro-porous structures with nano-grains of 5–40 nm and Ag NPs of <20 nm (1–4 µm)	Ti, anatase, rutile, and ZnO	1.06 (Ag)/22.19 (Zn) 1.42 (Ag)/26.93 (Zn) 1.56 (Ag)/29.38 (Zn)	Week 36 - - 684 (Ag)/6880 (Zn)	[87]
Cu and Zn										
CP-Ti	5	0.002 M CA, 0.02 M β-GP, (0, 0.005, 0.01, 0.02, and 0.04) M ZnA and 0.0013 M Cu(OAc)_2_	480	-	2	Micro-porous structures (1–4 µm)	Ti, anatase, and rutile	0.77 (Cu) 0.62 (Cu)/1.79 (Zn) 0.55 (Cu)/2.53 (Zn) 0.39 (Cu)/6.47 (Zn) 0.33 (Cu)/8.92 (Zn)	Day 20: 4.5 (Cu) 3.2 (Cu)/7.8 (Zn) 2.7 (Cu)/23.2 (Zn) 2.3 (Cu)/64.5 (Zn) 1.9 (Cu)/94.9 (Zn)	[77]
Ti6Al4V	9	3–9 g/L KOH, 5–11 g/L phytic acid, 2–10 g/L EDTA-CuNa_2_, 2–10 g/L EDTA-ZnNa_2_	-	11	3	Porous surface with increasing pore sizes for increased levels of Cu and/or Zn in surface	Ti, anatase	-/3.47 (Zn) -/9.84 (Zn) -/7.90 (Zn) 0.61 (Cu)/11.41 (Zn) 0.98 (Cu)/4.42 (Zn) 2.15 (Cu)/5.42 (Zn) -/5.64 (Zn) 1.25 (Cu)/6.71 (Zn) 4.18 (Cu)/2.89 (Zn)	-	[88]

CA: calcium acetate, Ca-GP: calcium glycerophosphate, GP: glycerophosphate, HA: hydroxyapatite, KOH: potassium hydroxide, NPs: nanoparticles, NR: not reported, SDBS: sodium dodecyl benzene sulfonate, TCP: tricalcium phosphate, ZnA: zinc acetate.

**Table 5:** Antibacterial tests and results on PEO-modified Ti-surfaces bearing single or multiple elements.

Bacterial Species	Bacterial Strain	Source	Analysis Method	Duration (h)	Test Inoculum	Planktonic/ Adherent	Main Outcomes	Ref.
Ag								
MRSA	AMC201	Ag NPs	Modified version of JIS Z 2801:2000	24	10^7^ CFU/mL	Adherent	After 24 h: 98 and 99.75% reduction by incorporation of 0.3 and 3 g/L Ag NPs	[15]
MRSA	AMC201	Ag NPs	Petrifilm^TM^ assay Zone of inhibition CFU count SEM Ex vivo	48	10^3^–10^8^ CFU/mL	Adherent	Significantly reduced numbers of viable bacterial colonies by incorporation of Ag NPs in the surface after 15 min. Four-logs reduction in the numbers of viable bacterial colonies in the ex vivo infection model by incorporation of Ag, compared with a 2-logs reduction in absence of Ag after 24 h. Prevention biofilm formation for at least 48 h	[31]
MRSA	AMC201	Ag NPs	Modified version of JIS Z 2801:2000	24	10^7^ CFU/mL	Adherent	100% killed by incorporation of 0.03wt% Ag at 24 h	[32]
*S. aureus* *E. coli*	ATCC6538 ATCC25922	AgNO_3_	Spread plate analysis	24	1.6∙10^5^ CFU/mL	Planktonic	After 24 h: >99.8 reduction by incorporation of >0.1 wt% Ag, compared with a reduction of 20% in absence of Ag	[35]
*E. coli*	ATCC25933	AgNO_3_	Spread plate analysis	12	10^6^ CFU/mL	Adherent	After 12 h: >99.9% eradication of *E. coli*	[48]
*S. aureus* *E. coli*	ATCC6538 ATCC25922	Ag NPs	CFU count Fluorescence measurement	24	0.0001 OD_590_	Adherent	After 24 h: complete eradication for *E. coli* and 6-log reduction for *S.aureus* with 5.8 at% Ag Stronger antibacterial effect on *E. coli* compared to *S. aureus*	[49]
MRSA	USA300	Ag NPs	Zone of inhibition CFU count SEM Ex vivo	48	10^4^–10^7^ CFU/mL	Adherent Planktonic	After 24 h: enhanced zone of inhibition for PT-AgSr samples compared to PT-Ag samples. Complete eradiation of adherent and planktonic bacteria in vitro and ex vivo. After 48 h: prevention of biofilm formation in Ag-containing surfaces.	[50]
*S.aureus* *E.coli*	NBRC122135 NBRC3972	AgNO_3_	ISO 22196:2007	24	0.4–3.0∙10^6^ CFU/mL	Adherent	After 24 h: >0.05 mM Ag in PEO electrolyte reduced bacteria >90%. Inhibitory effect was stronger for *E. coli* compared to *S. aureus*	[51]
*S. aureus*	209P	AgNO_3_	Spread plate analysis	2	500 CFU/mL	Planktonic	After 2 h: 53% reduction in CFU after incubation in supernatant	[52]
*S. aureus*	ATCC6538-P	AgNO_3_	Spread plate analysis	2	250 CFU/mL	Planktonic	After 2 h: 70% reduction in CFU and 45% antibacterial rate for >0.3%at Ag	[53]
*S. aureus*	NR	Ag_2_O	Spread plate analysis	24	10^5^ CFU/mL	Adherent	After 24 h: antibacterial rate >1 with 5.8wt% Ag	[54]
*S. aureus* *E. coli*	ATCC6538 ATCC25822	Ag-A	Spread plate analysis	24	2.5∙10^5^ CFU/mL	Planktonic	At 24 h: 99.9 and 58.3% reduction of E. coli for 4.6 wt% Ag and Ag-free. At 24 h: 99.8 and 47.8% reduction of S. aureus for 4.6 wt% Ag and Ag-free	[55]
*S. aureus*	ATCC6538	Ag-A	Modified version of JIS Z 2801:2000	24	2.5∙10^5^ CFU/mL	Planktonic	After 24 h: 99.98% reduction by incorporation of 1.14 wt% Ag	[56]
*S. mutans*	ATCC25175	Ag-A	Spread plate analysis SEM	16.5	1.5∙10^8^ CFU/mL	Adherent	After 16.5 h: 67% reduction by incorporation of 0.7 wt% Ag	[57]
*E. coli*	ATCC25822	AgNO_3_	Spread plate analysis	24	10^9^ CFU/mL	Planktonic	After 24 h: 97.4 and 99.2% reduction by incorporation of 0.6 and 2.1 wt% Ag, compared with a reduction of 22.7% in absence of Ag: Ag-free PEO-modified surface	[58]
*E. coli*	NBRC3972	AgNO_3_	ISO 22196:2011	24	5∙10^6^ CFU/mL	Planktonic	100% killed in presence of 0.01 wt% Ag at 24 h	[59]
*E. coli*	ATCC25922	Ag NPs	Spread plate analysis	24	10^6^ CFU/mL	Planktonic	100% killed by incorporation of 0.53 wt% Ag within 12 h	[60]
*S. sanguinis*	IAL1832	Ag NPs	Spread plate analysis	24	10^7^ CFU/mL	Planktonic	At 24 h: 62 and 53% reduction by incorporation of 1.9wt% Ag, compared to pure Ti and the Ag-free PEO-modified surface, respectively	[61]
*S. epidermidis*	ATCC35984	Ag NPs	Spread plate analysis SEM	18	10^6^ CFU/mL	Adherent Planktonic	100% killed by incorporation of 3.6at% Ag within 12 h	[63]
*P. gingivalis*	NR	Ag NPs	Microbial Viability Assay SEM	24	10^7^ CFU/mL	Adherent	Reduction of the bacterial viability to 21–31% by incorporation of <0.1wt% Ag at 8 h, compared with a mean viability of 96.6% in absence of Ag in the PEO-modified surface	[64]
*E. coli* *S. aureus* MRSA	ATCC25922 ATCC6538 Mu50	Ag-A	CFU count SEM	24	0.0005 OD_590_	Adherent	4–6 log inhibition of *E. coli*, 3–5 log inhibition of *S. aureus*, and 2–5 log inhibition of MRSA after 24 h for 0.1 and 0.5 and 0.8 g/L Ag respectively	[65]
*S. aureus*	B 918	Ag NPs	Spread plate analysis	24	10^6^ CFU/mL	Adherent	Lower amounts of adherent bacteria after 2 h. No inhibition at later time points	[66]
Cu								
*S. aureus*	NR	Cu(OAc)_2_	Spread plate analysis	4	10^6^ CFU/mL	Planktonic	Significantly reduced numbers of bacterial colonies by incorporation of 1.4 wt% Cu in the surface after 4 h	[36]
*S. aureus*	209P	Cu-substituted HA	Spread plate analysis	2	500 CFU/mL	Planktonic	After 2 h: 27% reduction in optical density after incubation in supernatant	[52]
*S. aureus*	NR	C_12_H_22_-CuO_14_	Spread plate analysis SEM	24	10^4^ CFU/mL	Adherent	After 24 h: 100% antibacterial rate on Cu surfaces Morphological changes and disrupted membrane of bacterial cells.	[67]
*S. aureus*	ATCC6538	EDTA-CuNa_2_	Live/dead staining SEM	24	10^5^ CFU/mL	Adherent	After 24 h: more dead bacteria on Cu surface compared to Ti control. Shape changes and membrane disruption of bacteria under SEM	[68]
*E. coli*	ATCC25922	Cu(NO_3_)_2_∙H_2_O	Zone of inhibition Adhesion test	24	10^8^ CFU/mL	Adherent Planktonic	After 24 h: zone of inhibition around 0.54–0.72 wt% Cu. No bacterial cells adhering after 24 h	[69]
*S. aureus* *E. Coli*	ATCC43300 ATCC25922	EDTA-CuNA_2_	Spread plate analysis	24	5∙10^5^ CFU/mL	Adherent	After 24 h: complete eradication of S. aureus and E. coli for 1.92 wt% Cu. After 14 days no antibacterial activity.	[70]
*S. aureus*	ATCC6538	CuA monohydrate	Spread plate analysis	24	10^5^ CFU/mL	Adherent	After 24 h: >99% growth reduction with 5.05 at% Cu in the surface.	[71]
*S. aureus*	ATCC25923	Cu(OAc)_2_	Spread plate analysis Live/dead staining SEM	96	10^5^ CFU/mL	Adherent Planktonic	At 6 h: 0.6 × 10^5^ CFU/cm^2^ on 1.93 wt% Cu-PEO and 1.5 × 10^5^ CFU/cm^2^ on Cu-free. At 24 h: 0.6 × 10^5^ CFU/cm^2^ on 1.93 wt% Cu-PEO and 9.7 × 10^5^ CFU/cm^2^ on Cu-free. At 6 h: 1.0 × 10^5^ CFU/mL for 1.93 wt% Cu- PEO and 3.8 × 10^5^ CFU/mL on Cu-free. At 24 h: 5.2 × 10^5^ CFU/mL for 1.93 wt% Cu-PEO and 200 × 10^5^ CFU/mL on Cu-free.	[72]
*S. aureus*	NR	CuSO_4_	Macrophage bactericidal assay SEM	2	10^7^ CFU/mL	Planktonic	Significantly enhanced macrophage-bactericidal capacity on 2 mM Cu-incorporated PEO-modified surface	[73]
*S. aureus* *E. coli*	NR	Cu NPs	Live/dead staining	24	10^5^ CFU/mL	Adherent	Majority of bacteria killed after 24 h	[74]
*S. aureus*	NR	Cu NPs	Spread plate analysis Live/dead staining SEM	24	10^7^ CFU/mL	Adherent Planktonic	100% killed by incorporation of 2.76 at% Cu at 24 h	[75]
*E. coli*	CMCC44102	Cu_2_O NPs	ASTM G21-13	24	NR	Adherent	At 24 h: 99.74% killed by incorporation of 10 g∙L-1 Cu_2_O NPs, compared to 95.25% killed in absence of Cu in the PEO-modified surface	[76]
Zn								
*S. aureus* *E. coli*	ATCC25923 ATCC25922	ZnO NPs	ASTM G21-1996	24	10^6^ CFU/mL	Planktonic	After 24 h: reduced numbers of viable colonies by incorporation of Zn compared with Zn-free surfaces	[37]
*S. aureus*	209P	Zn-substituted HA	Spread plate analysis	2	500 CFU/mL	Planktonic	After 2 h: 40% reduction in optical density after incubation in supernatant	[52]
*E. coli*	NR	ZnO NPs Zn-EDTA	Measurement of OD_600_	24	NR	Planktonic	After 24 h: 50% reduction in OD_600_ values of culture medium	[79]
*E. coli*	NBRC3972	ZnCl_2_	Spread plate analysis	24	4.9∙10^6^ CFU/mL	Adherent	After 24 h: less than 1 log reduction	[80]
*S. aureus* *E. coli*	ATCC25923 ATCC25922	ZnA	Spread plate analysis SEM	24	10^7^ CFU/mL	Planktonic	After 24 h: 40% enhanced antibacterial rate on *E.coli.* No effect on *S. aureus*	[81]
*S. aureus* *P. aeruginosa*	NR	ZnA	Live/dead staining SEM	24	OD_600_~0.35	Adherent	Significantly reduced numbers of viable colonies by incorporation of 9.7at% Zn at 6 and 24 h	[82]
*S. aureus* *E. coli*	ATCC25923 ATCC25922	ZnA	Spread plate analysis SEM	24	10^7^ CFU/mL	Adherent	At 24 h: 40.2, 99.2 and 100% reduction of *E. coli* for 4.6, 7.1, and 9.3 wt% Zn. At 24 h: 96.3, 99.5, and 99.8% reduction of *S. aureus* for 4.6, 7.1, and 9.3 wt% Zn	[83]
*S. aureus* *E. coli*	NR	ZnA	Spread plate analysis Live/dead staining SEM	24	10^5^ CFU/mL	Adherent Planktonic	>90% killed at 24 h	[84]
*S. mutans*	ATCC 25175	ZnA	Spread plate analysis SEM	48	10^9^ CFU/mL	Adherent	At 24 h: 62.54, 69.84 and 79.19% reduction for 0.199, 0.574 and 1.995at% Zn	[85]
*S. aureus* MRSA *S. epidermidis*	ATCC25923 MRSA1030 ATCC700296 *S. epidermidis* 15560	ZnO and Zn_3_(PO_4_)_2_ particles	Spread plate analysis	4	10^6^ CFU/mL	Adherent	After 4 h: no growth inhibition for *S. aureus* and MRSA, and 90% eradication of *S. epidermidis* on Zn-bearing surfaces.	[86]
Ag and Cu								
MRSA	USA300	Ag and Cu NPs	Zone of inhibition CFU count SEM Ex vivo	24	10^4^–10^7^ CFU/mL	Adherent Planktonic	After 24 h: zone of inhibition and eradication of adhering and planktonic bacteria in vitro and ex vivo for surface containing >50% Ag and Cu NPs. No antibacterial properties for solely Cu NP-bearing surfaces and controls.	[19]
Ag and Zn								
MRSA	USA300	Ag and Zn NPs	Zone of inhibition CFU count SEM Ex vivo	24	10^4^–10^7^ CFU/mL	Adherent Planktonic	After 24 h: zone of inhibition and eradication of adhering and planktonic bacteria in vitro and ex vivo for surface containing >50% Ag and Zn NPs. No antibacterial properties for solely Zn NP bearing surfaces and controls.	[20]
*S. aureus*	ATCC25923	Ag NPs and ZnA	Spread plate analysis SEM	24	10^5^ CFU/mL	Adherent Planktonic	At 24 h: 4.1, 2.5, and 2.4∙10^3^ CFU/cm^2^ on Ag and Zn co-doped surfaces compared with 2.3∙10^6^ CFU/cm^2^ on polished Ti, respectively. Significantly reduced numbers of viable colonies by incorporation of Ag NPs and Zn compared to polished Ti.	[87]
Cu and Zn								
*S. aureus*	ATCC25923	Cu(OAc)_2_ ZnA	Spread plate analysis Live/dead staining SEM	24	10^5^ CFU/mL	Adherent Planktonic	At 6 h: 2.63, 1.47, and 0.84∙10^5^ CFU/cm^2^ on Cu and Zn co-doped surfaces compared with 1.8, and 8.5∙10^5^ CFU/cm−2 on Cu-single doped and Cu-free surfaces, respectively. At 24 h: 3.72, 2.89, and 1.32∙10^5^ CFU/cm^2^ on Cu and Zn co-doped surfaces compared to 2.89 and 16∙10^5^ CFU/cm^2^ on Cu-single doped and Cu-free surfaces, respectively. Significantly reduced number of viable colonies by incorporation of >2.53 wt% Zn and <0.55 wt% Cu, compared to 0.77 wt% Cu	[77]
*E. coli*	CMCC44102	Cu_2_O and ZnO NPs	ASTM G21-13	24	10^6^ CFU/mL	Planktonic	PEO-modified surfaces bearing Cu_2_O NPs demonstrated a superior antibacterial activity~100% killed, compared with PEO-modified surfaces bearing ZnO NPs	[78]
MRSA *S. aureus* *E. coli*	ATCC43300 CGMCC12465 CGMCC13373	EDTA-CuNa_2_ EDTA-ZnNa_2_	Spread plate analysis	24	10^6^ CFU/mL	Adherent	After 24 h: complete prevention of growth with >6 g/L Cu or Zn in PEO electrolyte against MRSA, *S. aureus* and *E. coli*.	[88]

Ag-A: silver acetate, ASTM: American Society for Testing and Materials, CFU: colony forming unit, CuA: copper acetate HA; hydroxyapatite, JIS: Japanese Industrial Standards, NPs: nanoparticles, NR: not reported, SEM: scanning electron microscopy, ZnA: zinc acetate.

## Supplementary Materials

The following are available online at https://www.mdpi.com/article/10.3390/ijms22073800/s1, Figure S1: Flow diagram of the systematic literature search., Table S1: The biocompatibility of PEO-modified Ti-based surfaces bearing single or multiple elements.
