# Supplementary material for: Antibacterial Titanium Implants Biofunctionalized by Plasma Electrolytic Oxidation with Silver, Zinc, and Copper: A Systematic Review
Source: Int J Mol Sci. 2021 Apr 6;22(7):3800. doi: 10.3390/ijms22073800 (PMC8038786; doi:10.3390/ijms22073800)
Supplement: Supplementary file 1 [file ijms-22-03800-s001.zip › ijms-1131337-supplementary.docx]

| **Supplementatry Table 1.** The biocompatibility of PEO-modified Ti-based surfaces bearing single or multiple elements. | | |
| --- | --- | --- |
| Cell source | Main outcomes | Ref |
| Ag | | |
| Human osteosarcoma cell line (MG63 and HOS) | No adverse effects observed on the proliferation of MG63 cells and ALP activity of HOS cells by incorporation of <0.21% Ag: similar results were reported for the Ag-free surface.  Cytotoxic effect observed by incorporation of 0.21-0.45wt% Ag: no proliferation of MG63 cells. | [35] |
| Human osteosarcoma cell line (Saos-2) | No significant differences reported for cell proliferation by incorporation of 0.7wt% Ag, compared with the Ag-free surface, implying no cytotoxicity.  30% lower cell proliferation on the Ag-incorporated PEO-modified surface at day 7. | [57] |
|  | No cytotoxic effects observed by incorporation of 1.14 wt% Ag: no inhibition of cell viability.  Retarded cell proliferation characteristics observed on Ag-incorporated PEO-modified surfaces | [55] |
| Human osteoblastic cell line (SV-HFO) | No adverse effects observed on osteoblast viability and spreading activity by incorporation of 0.3 g∙L-1 Ag.  Cytotoxic effect observed by incorporation of 3 g/L Ag: low viability of SV-HFO cells. | [15] |
| Mouse pre-osteoblasts (MC3T3-E1) | Enhanced proliferation for 0.13at% Ag samples compared to bare Ti samples. | [48] |
|  | After 2 h no effect on cell seeding efficiency by Ag-bearing surfaces.  After 7 and 11 days no cytotoxic effects on PT-Ag surfaces, enhanced metabolic activity on PT-AgSr implants compared to control.  After 11 days no enhanced ALP activity on PT-Ag surfaces, enhanced ALP activity on PT-AgSr implants compared to control. | [50] |
|  | Reduction in cell number with > 2.5 mM Ag in PEO electrolyte.  No adverse effects on osteogenic differentiation and calcification. | [51] |
|  | No adverse effects observed on the cell adhesion and proliferation by incorporation of 1.6-5.8wt% Ag. | [54] |
|  | No adverse effects observed on the cell adhesion and proliferation by incorporation of 0.55at% Ag.  Incorporation of 0.69 and 0.80at% Ag in the PEO-modified surface retarded the cell proliferation rates. | [60] |
|  | No cytotoxicity by 0.1 and 0.5 g/L Ag-A and reduced cell viability with 0.8 g/L Ag-A after 24 h and 7 days. | [65] |
| Human mesenchymal stem cells (hMSCs) | No adverse effects observed on adhesion, spreading activity and proliferation by incorporation of 1.07at% Ag. | [61] |
|  | No cytotoxic effects observed by incorporation of 0.7wt% Ag NPs  Cell viability and cellular activities of the cells on Ag-bearing surfaces were comparable to Ag-free surfaces. | [62] |
|  | No cytotoxic effects observed by incorporation of 3.0 g/L Ag NPs.  No significant differences reported for cell viability, adhesion, and proliferation, compared with the Ag-free surface. | [31] |
| Mouse fibroblast cell line | No adverse effects observed on L929 cell morphology and viability by incorporation of <0.1wt% Ag. | [64] |
|  | >95% cell viability after direct 3T3-L1 cell culture for 24 h on coatings with 0.3-0.8wt% Ag. | [52] |
|  | >95% cell viability after direct L929 cell culture for 24 h on coatings with 0.2-0.8wt% Ag. | [53] |
| U2OS cell line | No cell toxicity on Ag surfaces  Enhanced collagen production after 2 weeks of culture on Ag surfaces | [66] |
| NR |  | [32, 49, 55, 58, 59, 63] |
| Cu | | |
| Human osteosarcoma cell line (MG63) | After 1, 3 and 7 days viability and cell activity for 1.92wt% Cu samples compared to control. | [70] |
|  | Adhesion, spreading, early proliferation and late differentiation were promoted by incorporation of 1.4wt% Cu.  No significant difference of ALP gene expression was observed.  COL-1 and OC gene expressions were significantly higher by incorporation of Cu in the PEO-modified surface.  Statically higher IGF-1 gene expression on Cu-incorporated PEO-modified surfaces, compared with Cu-free surfaces. | [36] |
| Mouse fibroblast cell line (L-929) | Adhesion, spreading and proliferation significantly increased on the PEO-modified surface with 0.67wt% Cu.  1.93wt% Cu induces cytotoxicity: mitochondrial activity and living number of L929-cells is about 50% of Ti  0.67wt% Cu up-regulated the extracellular collagen secretion compared to Cu-free modified-surface.  0.67-1.93wt% Cu up-regulated the production of intracellular specific protein contents: CTGF, α-SMA and COL-I: indicating an accelerated switch to fibrotic phenotype and differentiation to myofibroblasts. | [72] |
|  | Adhesion, spreading and proliferation significantly enhanced on Cu-bearing surfaces (0.77wt%)  Significantly enhanced extracellular collagen secretion and production of intracellular specific proteins: CTFG and COL-I, by incorporation of 0.77wt% Cu: indicating an accelerated switch to fibrotic phenotype.  No cytotoxic effects observed by incorporation of 0.77wt% Cu. | [77] |
|  | >95% cell viability after direct L929 cell culture for 24 h on coatings with 0.1-0.4wt% Cu. | [52] |
| Human osteosarcoma cell line (Saos-2) and murine macrophages | Significantly enhanced macrophage proliferation on the PEO-modified surface incorporated with 2 mM Cu.  Cu-bearing surface induced higher expression levels of iNOS and lower levels of Arg1.  The release concentrations of pro-inflammatory cytokine IL-6 were significantly higher, contradictory to anti-inflammatory cytokines Il-4 and IL-10: indicates macrophage polarization to M1 phenotype.  Macrophages on Cu-bearing surface induced a favorable osteogenic inflammatory microenvironment: extracellular collagen secretion and ECM mineralization by Saos-2 cells were significantly enhanced. | [73] |
| Mouse pre-osteoblasts (MC3T3-E1) | No cytotoxicity for Cu surfaces compared to Ti controls after 24 h.  Increased number of adherent cells compared to Ti controls.  Enhanced proliferation rates compared to Ti controls.  Increased expression of osteogenic genes.  Reduced number of apoptotic cells. | [67] |
|  | High level of viable cells on brown area compared to black area after 24 h.  Osteogenic differentiation as measured by ALP activity enhanced. | [68] |
|  | Enhanced cell attachment on surface compared to bare Ti specimens.  Reduced spreading of cells on Cu specimens after 24 h.  Cytotoxic effect for Cu specimens after 24 h. | [71] |
|  | 1.3at% Cu promoted MT3T3-E1 cell adhesion, proliferation and viability.  Incorporation of 1.3at% Cu in PEO-modified surface enhanced ECM mineralization.  Cytotoxic effect observed by incorporation of 2.76at% Cu: low viability of MT3T3-E1 cells.  No adverse effects observed on ALP activity and extracellular collagen secretion.  Cu-bearing surfaces favor adhesion, spreading, and proliferation of endothelial cells, without cytotoxicity.  Cu-bearing surfaces up-regulated the secretion of VEGF and production of NO, positively related to the Cu concentration in the surface. | [75] |
| NR |  | [69, 74, 76, 78] |
| Zn | | |
| Adipose-derived stem cells (ADSC) | No adverse effects observed on cell adhesion and proliferation by incorporation of 9.7at% Zn: comparable results with the Zn-free PEO-modified surface. | [82] |
| Rat bone marrow stem cells (bMSC) | Initial adhesion, spreading activity, proliferation and differentiation were significantly enhanced on Zn-incorporated PEO-modified surfaces compared with Zn-free surfaces, implying no cytotoxic effects.  ALP activity was significantly elevated on the Zn-incorporated PEO-modified surface: concentration-dependent. | [83] |
| Human osteosarcoma cell line (MG63) | Cell proliferation and ALP activity significantly enhanced by incorporation of 0.199-0.574at% Zn in surfaces.  Cytotoxic effect observed by incorporation of 1.995at% Zn: no viable cells observed on surface.  Bone-cell activity negatively related to the concentration of Zn within limits. | [85] |
|  | Zn-bearing surfaces enhanced proliferation and viability with 10-20% after 7 days. | [86] |
| Mouse pre-osteoblasts (MC3T3-E1) | No cytotoxic effect of Zn surfaces compared to Ti specimens after 1-3 days.  Calcification of Zn surfaces enhanced compared to Ti specimens, however not to non-Zn PEO controls. | [80] |
| Mouse fibroblast cell line (L-929) | >95% cell viability after direct L929 cell culture for 24 h on coatings with 0.1-0.4wt% Zn. | [52] |
| NR |  | [37, 79, 81, 84] |
| Ag and Cu | | |
| Mouse pre-osteoblasts (MC3T3-E1 | Enhanced metabolic activity after 7 and 11 days for Cu NP bearing surfaces compared to Ag and Cu NP bearing surfaces.  No cytotoxic effects of Ag NP bearing surfaces.  No enhanced ALP activity of biofunctionalized surfaces with or without Ag and/or Cu NPs compared controls. | [19] |
| Ag and Zn | | |
| Mouse pre-osteoblasts (MC3T3-E1) | Enhanced metabolic activity after 7 and 11 days for Zn NP bearing surfaces compared to Ag and Zn NP bearing surfaces.  No cytotoxic effects of Ag NP bearing surfaces.  No enhanced ALP activity of biofunctionalized surfaces with or without Ag and/or Zn NPs compared controls. | [20] |
| NR |  | [87] |
| Cu and Zn | | |
| Mouse fibroblast cell line (L-929) | Adhesion, spreading activity and proliferation significantly enhanced on co-doped PEO-modified surfaces with Cu and Zn, compared with Cu single-doped PEO-modified surfaces.  Co-doped PEO-modified surface with Cu (0.55-0.39wt%) and Zn (2.53-6.47wt%) improved the extracellular collagen secretion and the production of intracellular specific protein: CTGF and COL-I, compared with the Cu single-doped surface (0.77wt%).  No stimulatory affects observed on co-doped surfaces with 0.62wt% Cu and 1.79wt% Zn.  Cytotoxic effects observed by incorporation of 0.33wt% Cu and 8.92wt% Zn: low viability of L929 cells. | [77] |
| Human osteosarcoma cell line (MG63) | After 4 h enhanced cell attachment on Cu and/or Zn bearing surfaces compared to Ti control.  After 1 and 3 days reduced proliferation, while after 7 days enhanced proliferation of Cu and/or Zn bearing surface compared to Ti control. | [88] |
| ADSC: adipose derived stem cells, ALP: alkaline phosphatase activity, Arg1: arginase1, bMSC: bone marrow stem cells, COL-I: type I collagen, CTGF: connective tissue growth factor, ECM: extracellular matrix, HOS: human osteosarcoma, IGF-1: insulin-like growth factor-1, IL: interleukin, iNOS: inducible nitric oxide synthase, NO: nitric oxide, NPs: nanoparticles, NR: not reported, OC: osteocalcin, PEO: plasma electrolytic oxidation, α-SMA: alpha smooth muscle action, SV-HFO: simian virus human fetal osteoblasts, VEGF: vascular endothelial growth factor. | | |
