# Supplementary figures and images for: Antibacterial Titanium Implants Biofunctionalized by Plasma Electrolytic Oxidation with Silver, Zinc, and Copper: A Systematic Review
Source: Int J Mol Sci. 2021 Apr 6;22(7):3800. doi: 10.3390/ijms22073800 (PMC8038786; doi:10.3390/ijms22073800)

Suppl.figure 1

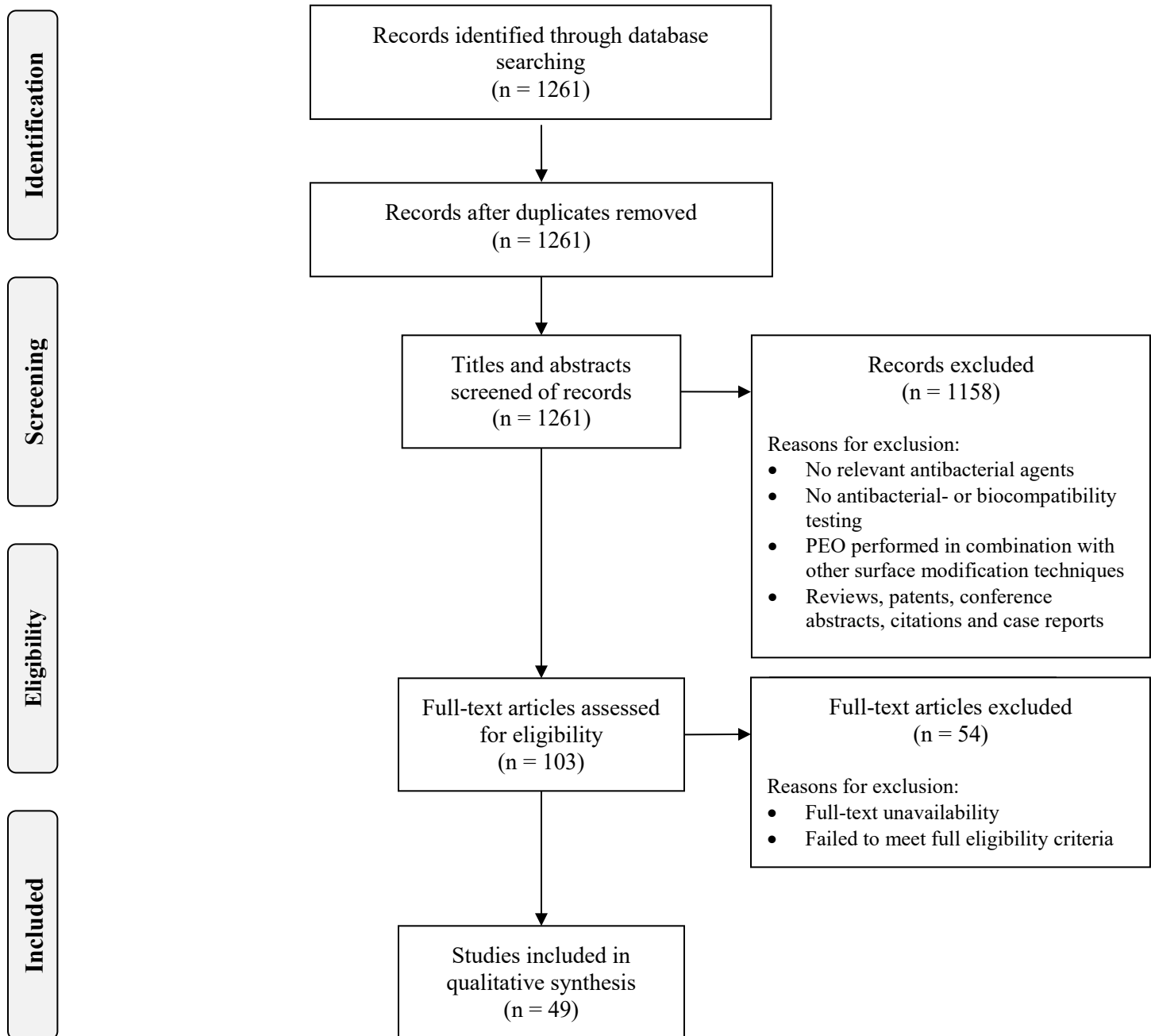

Supplement: Supplementary file 1 [file ijms-22-03800-s001.zip › ijms-1131337.pdf]
